# The dynamic arms race during the early invasion of woodland strawberry by *Botrytis cinerea* revealed by dual dense high-resolution RNA-seq analyses

**DOI:** 10.1093/hr/uhad225

**Published:** 2023-11-09

**Authors:** Yibo Bai, Haibin Wang, Kaikai Zhu, Zong-Ming Cheng

**Affiliations:** College of Horticulture, Nanjing Agricultural University, Nanjing 210095, China; Key Laboratory of Crop Gene Resources and Germplasm Enhancement in Southern China, Ministry of Agriculture; Tropical Crops Genetic Resources Institute, Chinese Academy of Tropical Agricultural Sciences, Haikou 571101, China; College of Horticulture, Nanjing Agricultural University, Nanjing 210095, China; Co-innovation Center for Sustainable Forestry in Southern China, Nanjing Forestry University, Nanjing, Jiangsu 210037, China; College of Horticulture, Nanjing Agricultural University, Nanjing 210095, China

## Abstract

Necrotrophic pathogens replicate massively upon colonizing plants, causing large-scale wilting and death of plant tissues. Understanding both mechanisms of pathogen invasion and host response processes prior to symptom appearance and their key regulatory networks is therefore important for defense against pathogen attack. Here, we investigated the mechanisms of interaction between woodland strawberry (*Fragaria vesca*) leaves and gray mold pathogen (*Botrytis cinerea*) at 14 infection time points during the first 12 hours of the infection period using a dense, high-resolution time series dual transcriptomic analysis, characterizing the arms race between strawberry *F. vesca* and *B. cinerea* before the appearance of localized lesions. Strawberry leaves rapidly initiated strong systemic defenses at the first sign of external stimulation and showed lower levels of transcriptomic change later in the infection process. Unlike the host plants, *B. cinerea* showed larger-scale transcriptomic changes that persisted throughout the infection process. Weighted gene co-expression network analysis identified highly correlated genes in 32 gene expression modules between *B. cinerea* and strawberry. Yeast two-hybrid and bimolecular fluorescence complementation assays revealed that the disease response protein FvRLP2 from woodland strawberry interacted with the cell death inducing proteins BcXYG1 and BcPG3 from *B. cinerea*. Overexpression of *FvRLP2* in both strawberry and *Arabidopsis* inhibited *B. cinerea* infection, confirming these genes’ respective functions. These findings shed light on the arms race process by which *B. cinerea* invades host plants and strawberry to defend against pathogen infection.

## Introduction

In nature, plants inhabit environments that bristle with microorganisms and are subjected to biotic and abiotic stresses throughout their growth and development [1, 2]. Pathogens may derive nutrients from living tissue by invading host cells (biotrophy) or from dead tissue by killing cells and feeding on their contents (necrotrophy) [3, 72]. Compared with biotrophic fungi, necrotrophic pathogens pose a greater threat to plant production, and the diseases they cause inflict significant economic losses [4]. Necrotrophic pathogens attack the plant’s defense system by hijacking the disease resistance genes in the host [5, 6]. For example, the *Phytophthora capsici* effector *RxLR207* has been reported to activate reactive oxygen species (ROS)-mediated cell death in *Arabidopsis* by targeting *BPA1* and *BPLs*, which participated in programmed cell death and defense responses [7]. *PIAvh142* from *Peronophythora litchii* trigger cell death in several plant species through a process dependent on the signaling components *RAR1*, *SGT1*, and *HSP90* [8].

*Botrytis cinerea* (Bc05.10) is one of the most widespread necrotrophic fungal pathogens, it causes gray mold in over 200 plant species, including many important crop plants like strawberry, grape, tomato, pepper, etc., causing US$ 10 to 100 billion annual economic loss worldwide [9]. *B. cinerea* has evolved multiple strategies for attacking plants [10]. It secretes metabolites, small RNAs, and extracellular proteins to kill the host [11–13]. Additionally, Phytotoxic necrosis- and ethylene-inducing peptide (Nep1)-like proteins are involved in callose deposition, reactive oxygen species formation, and ethylene accumulation in the host [14]. Pectic polysaccharides in plant cell walls were hydrolyzed by endopolygalacturonases (PGs) produced by *B. cinerea* [15], and its xyloglucanase (*BcXYGs*) was recognized as microbe-associated molecular patterns by the leucine-rich repeat receptor-like kinases BAK1 and SOBIR1 [16].

Plants rely on their innate immune system to respond to external infection by pathogens such as *B. cinerea* [17], and previous studies have shown that the majority of related gene expression occurred before the development of overt lesions [18]. Activation of host defense mechanisms in response to certain concentrations of *B. cinerea* conidia was accompanied by enhancement of plant cell wall structure and local production of disease-related proteins and reactive oxygen species [19]. High-resolution temporal transcriptomic analysis has revealed detailed changes in the expression of various *Arabidopsis* genes in response to *B. cinerea* before the appearance of lesions [20]. However, so far, there is lack of detailed characterization of interactions between *B. cinerea* and host plants at early infection stages, particularly in economically relevant, non-model plant species. High-resolution temporal RNA-seq of both pathogen and host plant could be used to study early interactions between *B. cinerea* and its host in detail, providing guidance for the development of efficient control strategies and resistant plant varieties.

A dual RNA-seq approach has been used to study a limited number of plant–pathogen interactions [21–23]. New *Aspergillus flavus* resistance genes were identified in maize by constructing an *A. flavus*-maize regulatory network [24]. Likewise, dual RNA-seq revealed potential mechanisms of interaction between *Chrysanthemum morifolium* and *Alternaria alternata*, laying the foundation for the development of new disease-resistant chrysanthemum varieties [25]. However, these studies were conducted during the entire infection process until the severe symptom development, and RNA-seq data were collected in long intervals, in days, rather than in hours.

Strawberry is a worldwide important crop and is highly susceptible to *B. cinerea* both in leaf and fruit tissues. Once exposed to the conidia of *B. cinerea*, it can cause the above-ground tissue of strawberry to decay, which affects the quality and yield of the strawberry. In severe cases, it can even lead to the death of the whole plant. According to statistics, gray mold caused by *B. cinerea* can lead to a 60% reduction in strawberry production and has become one of the major diseases that threaten strawberry cultivation [26]. To date, there have been few studies on the early interaction between *Fragaria vesca* (woodland strawberry) and *B. cinerea*. Here, we performed the simultaneous dense transcriptomic analysis of *F. vesca* and *B. cinerea* during the first 12 hours of infection with RNA-seq interval in hours, identifying differentially expressed genes in both pathogen and host plant during infection. Weighted gene co-expression network analysis (WGCNA) identified genes whose expression patterns were highly correlated between the pathogen and host, providing insight into the attack mechanisms of *B. cinerea* and the defense mechanisms of *F. vesca*. We documented the interaction between the fungal secreted proteins BcXYG1 and BcPG3 and the resistance proteins FvRLP2 and showed that overexpression of the *FvRLP2* gene inhibited *B. cinerea* infection of strawberry and *Arabidopsis*. Our findings provided new insights for the prevention of *B. cinerea* and the potential for molecular breeding of strawberry cultivars with improved pathogen resistance, thereby reducing economic losses caused by *B. cinerea*.

## Results

### Early phenotypic characteristics of woodland strawberry infected by *B. cinerea*

**Figure 1:**
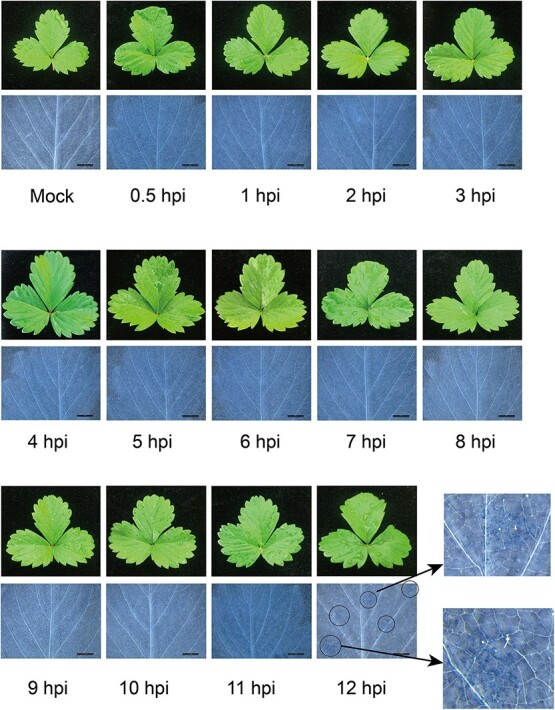
Phenotypic observation of woodland strawberry at treatment points, the length of the line in the figure represents 5 mm. The upper row showed the changes in strawberry leaves at 0–12 hpi. The lower row showed the occurrence of lesions visualized by trypan blue staining, and lesions appeared at 12 hpi, as indicated by circles.

Compared with the control strawberry leaves, there was no significant change in leaves at 0.5–11 hpi. However, at 12 hpi, dead cells were observed in the strawberry leaves by trypan blue staining (Fig. 1). We identified the 0–12 hpi as the early stage of infection with *B. cinerea* in strawberry.

The activity of antioxidant enzymes (POD and CAT) and the content of H_2_O_2_ and MDA were measured to understand the detailed changes in the early stage of *B. cinerea* infection in strawberry (Fig. S1, see online supplementary material). With the processing time prolonged, the content of MDA gradually increases from 40 mmol/g at 0 hpi, especially during the 11–12 hpi, the content of MDA increases from 78 mmol/g to 97 mmol/g. The content of H_2_O_2_ increased from 6.6 μmol/g increased to 9.4 μmol/g, with a significant increase mainly in the 11–12 hpi. The activity of CAT increased from 0.5 U/g at 0 hpi to 10.3 U/g at 12 hpi. The activity of POD showed a zigzag upward trend, from 285 U/g at 0 hpi to 694 U/g at 12 hpi. These results indicated that in the asymptomatic infection stage, strawberry respond to changes in the external environment to enhance stress resistance.

### Dual transcriptome analysis during early infection of *B. cinerea*

**Figure 2:**
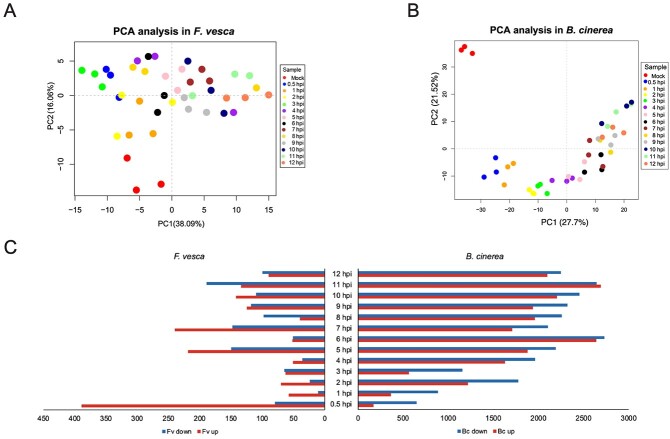
Differentially expressed genes analysis in *Botryti cinerea* and *Fragaria vesca* throughout the time course of early infection. (**A**) PCA analysis of each time point in *F. vesca*. (**B**) PCA analysis of each time point in *B. cinerea*. (**C**) Numbers of up-and down-regulated DEGs (inoculated vs. control) at different time points after inoculation.

The simultaneous RNA-seq analysis of leaf and fungal tissues on 14 time points at 0 hpi to 12 hpi, with three biological replicates per treatment, resulted in a total of 84 libraries (Fig. S2, see online supplementary material). Clean reads were aligned to the genomes of *F. vesca* [27] and *B. cinerea* [28]. Together, the mixed leaf/fungus samples (0.5–12 hpi) and the *F. vesca* control sample yielded 644.53 Gb of clean data, 96.53% of the *F. vesca* control reads mapped to the *F. vesca* genome, and between 95.78% and 66.4% of the reads from individual mixed samples mapped to the *F. vesca* genome (Table S1, see online supplementary material). Similarly, the mixed leaf/fungus samples (0.5–12 hpi) and the *B. cinerea* control sample yielded 638.06 Gb clean data, and 97.84% of the reads from the fungal control sample and 1.23% at 0.5 hpi to 31.32% at 12 hpi of the reads from individual mixed samples mapped to the *B. cinerea* genome (Table S2, see online supplementary material). The percentage of mixed-sample reads that mapped to the *B. cinerea* genome increased as the infection progressed. The PCA analysis based on DEGs-related TPM values identified that the replicates had close clustering between each time point, and there was a significant difference between the treated samples and the control samples (Fig. 2A and B).

The eight genes of *F. vesca* and *B. cinerea* were randomly selected for qRT-PCR analysis to verify the reliability of transcriptome data (Fig. S3, see online supplementary material). The expression patterns of RNA-seq and qRT-PCR data of eight genes showed high consistency. These results indicated obtained high-quality RNA-seq data.

### Characteristics of DEGs during the early stages of infection

A total of 1254 differentially expressed genes (DEGs) from 14 treatment points were identified in *F. vesca* leaves across the infection time points (|log_2_FC| ≥ 1, FDR < 0.05), including 843 upregulated and 411 downregulated DEGs (Fig. 2C, Table S3). The largest number of upregulated strawberry genes were identified at 0.5 hpi. A total of 7074 DEGs were identified in *B. cinerea* across the 12 h infection course, including 3369 upregulated and 3705 downregulated DEGs (Fig. 2C, Table S4). Compared with *F. vesca*, *B. cinerea* had more DEGs, especially later in infection, which may reflect the key role of these genes in the progression of infection.

**Figure 3:**
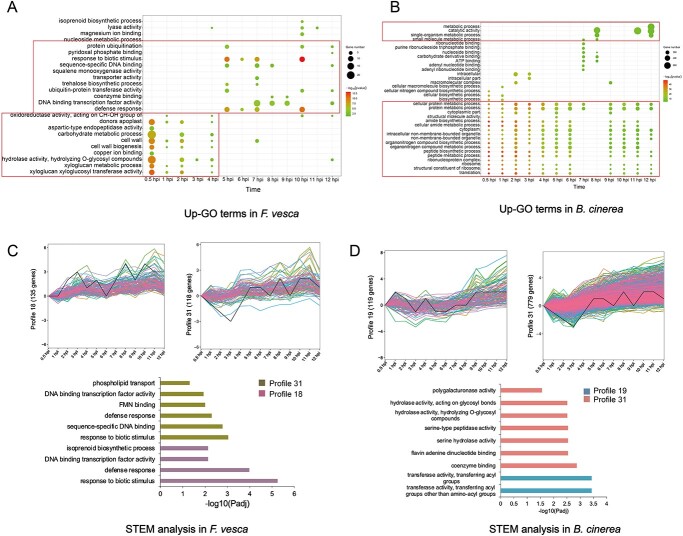
Expression dynamics of DEGs in *Fragaria vesca* and *Botryti cinerea* throughout the time course of early infection. (**A**) GO functional enrichment analysis of upregulated DEGs in *F. vesca*. (**B**) GO functional enrichment analysis of upregulated DEGs in *B. cinerea*. (**C**–**D**) Significantly enriched GO terms in significant expression time course profiles of the DEGs from *F. vesca* (**C**) and *B. cinerea* (**D**).

The DEGs were further characterized by GO enrichment (Fig. 3A and B; Fig. S4, see online supplementary material). Among the upregulated strawberry genes, GO terms such as ‘xyloglucan xyloglucosyl transferase activity’, ‘carbohydrate metabolic process’ and ‘cell wall biogenesis’ were highly enriched at the initial stage of infection (0.5–4 hpi). Additional highly enriched GO terms included ‘defense response’ and ‘response to biotic stimulus’ at 5–7 hpi. ‘DNA binding transcription factor activity’ at 7–9 hpi and ‘ubiquitin-protein transferase activity’ and ‘protein ubiquitination’ at 10–12 hpi. Among the upregulated *B. cinerea* genes, numerous GO terms related to translation, metabolic process and biosynthetic process were highly enriched at the early stages of infection. In contrast to the more varied patterns of GO enrichment in *F. vesca*, many more enriched GO terms in *B. cinerea* remained consistent throughout the infection time course, perhaps reflecting processes required for the sustained attack of the host by the necrotrophic pathogen.

The temporal expression profiles of DEGs from *F. vesca* and *B. cinerea* were analysed across 13 infection time points. In *F. vesca*, six expression profiles had statistically significant genes (*P* ≤ 0.01) (Fig. 3C; Fig. S5A, see online supplementary material). Profiles 18 and 31 contained 134 and 118 DEGs, respectively, and each showed a slightly different pattern of upregulation during infection (Table S5). Genes in profile 18 were initially upregulated, then downregulated, and increased in expression thereafter. Genes in profile 31 were strongly downregulated early in infection, then upregulated later, with some variation at individual time points. Genes in these two profiles were significantly enriched in the GO terms ‘response to biotic stimulus’, ‘defense response’ and ‘DNA binding transcription factor activity’, suggesting that these DEGs played a role in pathogen response (Fig. 3C). In *B. cinerea*, six of which were assigned a statistically significant number of genes (*P* ≤ 0.01) (Fig. 3D; Fig. S5B, Table S6, see online supplementary material). Genes in these six profiles showed fluctuating patterns of upregulation, and GO analysis revealed that they were enriched in aspects of protein catabolism and hydrolase activity.

### Expression patterns of genes involved in defense in *F. vesca*

**Figure 4:**
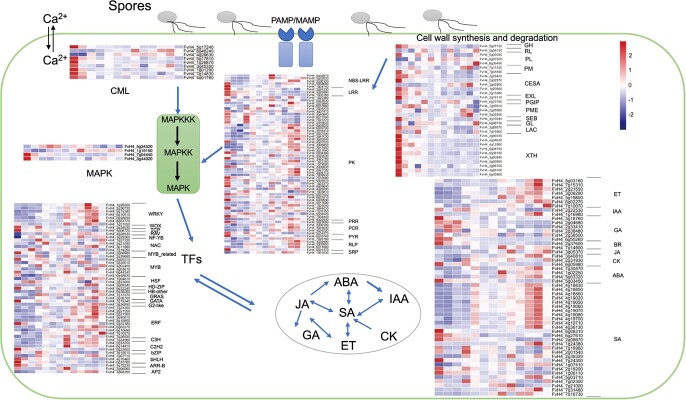
Expression heatmaps of *Fragaria vesca* genes involved in cell wall synthesis and degradation, hormone signaling, pathogen recognition and signaling, and transcriptional regulation (i.e. transcription factors) over a 12-h time course after *Botryti cinerea* inoculation.

The epidermal cuticle and cell wall were the first line of plant defense against pathogen infection [29]. Numerous genes related to cell wall synthesis and degradation were highly expressed in the early stage of infection. For instance, *FvH4_2g29420* (CESA) was highly expressed at 0.5 hpi and 1 hpi, *FvH4_7g16960* (LAC) was highly expressed at 3 hpi, and *FvH4_7g11320*, *FvH4_3g42900* (CESA) and *FvH4_5g35590* (PMEIs) were most highly expressed at 11 hpi (Fig. 4). We found the highly expressed pattern recognition receptor PRR (*FvH4_5g23690*), which was related to microbe-associated molecular patterns, at the initial stage of infection (0.5 hpi) and upregulated again at 9–11 hpi (Fig. 4). LRR genes (*FvH4_2g24330*, *FvH4_3g20490* and *FvH4_1g05180*) associated with the effector-triggered immunity, were highly expressed mainly at 0.5 hpi. However, intracellular nucleotide-binding (NB) LRR domain receptor (NBS-LRR) genes were most strongly expressed at 7 hpi (*FvH4_5g32810*) and 10–12 hpi (*FvH4_5g32820* and *FvH4_2g36100*). TFs participate in plant defense against pathogens through multiple pathways [30, 31]. In this study, 22 TF families were found to be expressed during the early stage of *B. cinerea* infection, including well-known WRKY, MYB, ERF, C2H2, bZIP, and bHLH family members. The expression levels of WRKY, ERF, C3H, and C2H2 transcription factor family members were higher at 7–11 hpi. In addition, WOX, TCP, and RAV gene family members were up-regulated at 3 hpi and 4–11 hpi, respectively. Hormones have been shown to act as central signaling molecules in plant defense against pathogen [32, 71]. Genes associated with ethylene, auxin, gibberellin, brassinolide, jasmonic acid, cytokinin, abscisic acid, and salicylic acid were expressed during the asymptomatic infection period (Fig. 4). Genes related to the ethylene pathway were mainly expressed later in infection at 8–12 hpi. A large number of salicylic acid pathway genes were expressed at various times throughout the infection period.

### Analysis of pathogenesis genes in *B. cinerea*

**Figure 5:**
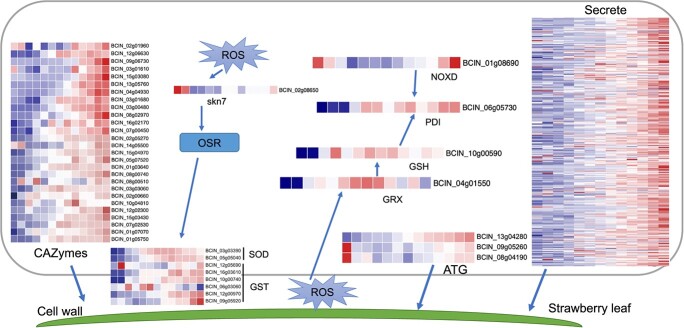
Expression heatmaps of DEGs related to the virulence of *Botryti cinerea* over a 12-h time course after inoculation onto *Fragaria vesca* leaves.

The evolution of defense pathways in plants accelerates selection processes on microbial pathogens to escape plant immune recognition [33]. We found that the expression of the autophagy-related gene *BCIN_13g04280* (*BcAtg18*) increased gradually from 7 hpi to 12 hpi, and expression of *BCIN_09g05260* (*BcAtg5*) and *BCIN_08g04190* (*BcAtg16*) was high, mainly at 0.5 hpi and 11–12 hpi (Fig. 5). In addition, the glutathione S-transferase genes *BCIN_12g05690* (*Bcgst20*) and *BCIN_06g03060* (*Bcgst9*) were highly expressed at 0.5 hpi and 2–5 hpi, respectively (Fig. 5). CAZymes in *B. cinerea* promoted host penetration by degrading pectin in the plant cell wall. In this study, the genes encoding glucosyltransferase (*BcIN_02g00660* and *BcIN_01g07070*) were highly expressed at 3 hpi. Most CAZymes were highly expressed after 5 hpi. In the early stage of infection, *B. cinerea* forms its own ROS to promote oxidative burst in plants. The *skn7* (*BCIN_0208650*) gene associated with oxidative stress-responsive was highly expressed at 0.5–1 hpi. Genes of *GRX* (*BCIN_04g01550*), *GSH* (*BCIN_10g00590*), *PDI* (*BCIN_06g05730*) were expressed after 2 hpi. As an early cell death inducing factor, secreted proteins play a key role in the early local necrosis of plants. In this study, we found that the expression levels of many genes encoding secretory proteins gradually increased, and most of them increased with the extension of infection time after 5 hpi (Fig. 5, Table S7). For example, *BCIN_03g03630* (*BcXYG1*) and *BCIN_13g03350* genes encoding xyloglucan-specific β-1,4-glucanase were up-regulated after 2 hpi, and the expression levels were increased with the extension of treatment time. In general, the expression of pathogenesis genes in *B. cinerea* increased with increasing infection time.

### Co-expression of genes from *F. vesca* and *B. cinerea*

**Figure 6:**
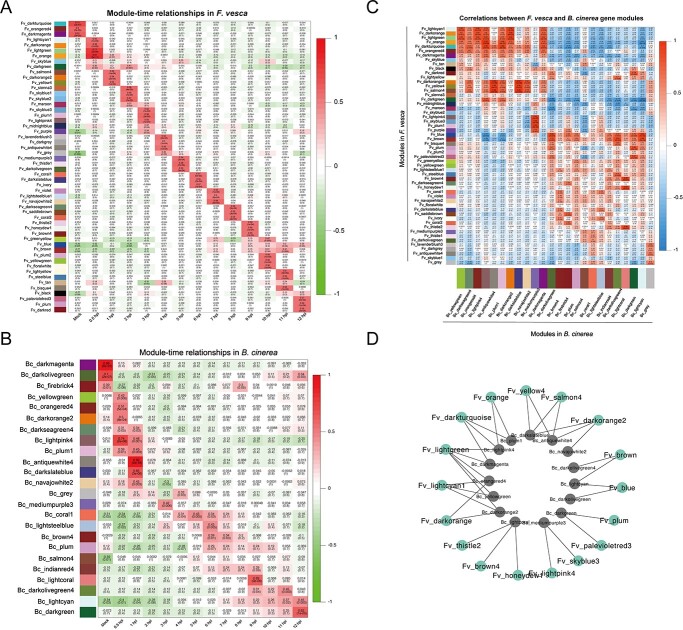
WGCNA results revealed gene modules that were highly correlated with expression time points in *Fragaria vesca* (**A**) and *Botryti cinerea* (**B**).(**C**) Correlation analysis between WGCNA modules from *F. vesca* and *B. cinerea*. (**D**) Network of *F. vesca* and *B. cinerea* modules (*r* ≥ 0.8 and *P*-value <0.05).

Weighted gene co-expression network analysis (WGCNA) was used to identify co-expressed gene modules in *F. vesca* (Fig. 6A) and *B. cinerea* (Fig. 6B) at different infection time points. A total of 29 958 genes were divided into 54 co-expression modules, and modules and infection stages showed a significant positive correlation during infection (Fig. 6A). Through KEGG pathway annotation of the genes in these significant expression modules, the enrichment pathways from the genes in the significant expression modules in each stage were determined (Fig. S6A, see online supplementary material). ‘cAMP signaling pathway’, ‘glycerophospholipid metabolism’, ‘biosynthesis of cutin, lignan and wax’, ‘plant hormone signal transduction’ and ‘plant-pathogen interaction’ were enriched at 0.5 hpi, indicating that plants can quickly perceive external changes and make stress responses. The pathways related to oxidative phosphorylation and CYP450 metabolism were mainly enriched at 1 hpi. It mainly enriched various signal transduction, biosynthesis and metabolic pathways at 2–5 hpi. Starch and sucrose metabolic pathways were mainly enriched at 6 hpi. MAPK signaling pathway and metabolic and biosynthetic pathways were enriched at 7 hpi. The glycolysis pathway was mainly enriched at 8 hpi. It was mainly enriched in the biosynthesis of phenylalanine at 9 hpi. Protein hydrolysis, autophagy and various biosynthesis and metabolic pathways were mainly enriched at 10–12 hpi.

A total of 12 311 genes from *B. cinerea* were divided into 12 co-expression modules, respectively (Fig. 6B). The pathways related to ribosome biogenesis and amino acid biosynthesis were enriched in the co-expression modules at 0.5 hpi (Fig. S6B, see online supplementary material). It mainly enriched various metabolic activity pathways at 1 hpi. The pathways related to secondary metabolism and biosynthesis were mainly enriched at 4–5 hpi. In addition, we also found an autophagy-related pathway at 4–5 hpi, which is related to the virulence and pathogenicity of *B. cinerea*. The pathways related to starch and sucrose metabolism were mainly enriched at 6–7 hpi. Pathways related to fatty acid metabolism and biosynthesis were mainly enriched at 9 hpi, indicating that *B. cinerea* was attacking the host surface at this time. The pathways of protein processing, N-glycan biosynthesis and N-glycine biosynthesis were mainly enriched at 10–12 hpi. The enrichment of these pathways indicated that *B. cinerea* was constantly absorbing the plant’s sugar substances to attack the host.

### Interactions of the gene modules among *F. vesca* and *B. cinerea*

**Figure 7:**
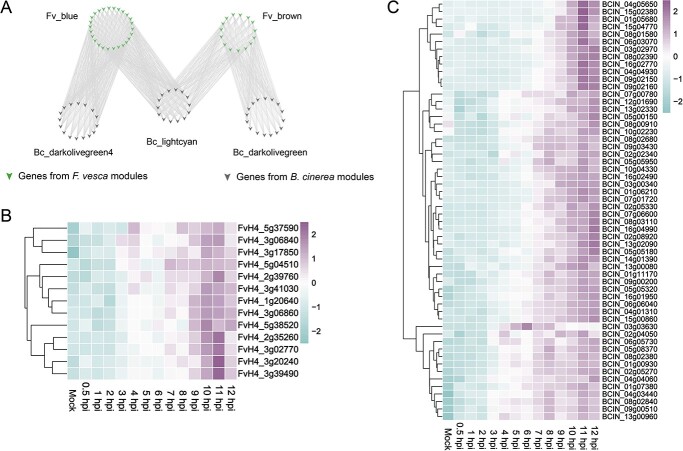
Interaction analysis between *Fragaria vesca* and *Botryti cinerea*. (**A**) Gene network of highly correlated gene modules from *F. vesca* and *B. cinerea* at 11–12 hpi (*r* ≥ 0.9 and *P*-value <0.05). (**B**) Heatmap of highly correlated *RLP* genes at Fv_brown module from *F. vesca*. (**C**) Heatmap of highly correlated effector genes at Bc_lightcyan module from *B. cinerea*.

**Figure 8:**
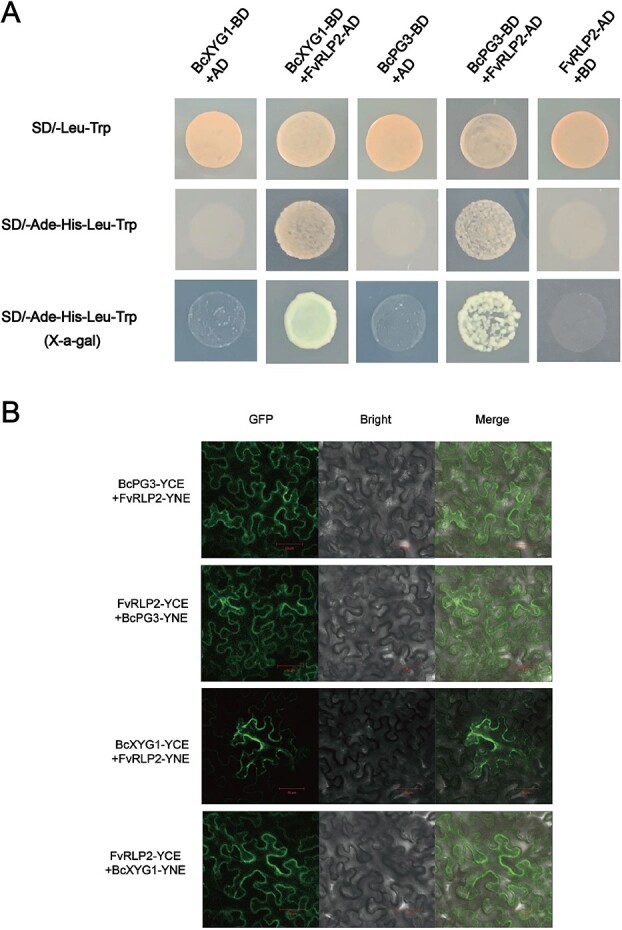
Physical interactions between highly correlated genes in *Botryti cinerea* and *Fragaria vesca*. (**A**) Interactions between *BcPG3*, and *BcXYG1* from *B. cinerea* and *FvPLR2* from *F. vesca* were demonstrated by yeast two-hybrid assays. *BcPG3* and *BcXYG1* were cloned into the bait plasmid pGBKT7, and *FvPLR2* was cloned into the prey plasmid pGADT7. (**B**) Interactions between *BcPG3* and *BcXYG1* from *B. cinerea* and *FvPLR2* from *F. vesca* were demonstrated in a bimolecular fluorescence complementation (BiFC) assay. Confocal laser scanning microscopy was used to observe the fluorescence signal. Bars = 50 μm.

To explore the interaction between *B. cinerea* and *F. vesca*, WGCNA was used to perform a correlation analysis among gene modules from the two species (Fig. 6C). There were high, significant correlations among 17 modules from strawberry and 15 modules from *B. cinerea* (*r* ≥ 0.8 and *P*-value <0.05) (Fig. 6D). Five *F. vesca* modules (Fv_orange, Fv_darkturquoise, Fv_lightgreen, Fv_lightcyan1, and Fv_darkorange) and six *B. cinerea* modules (Bc_plum1, Bc_lightpink4, Bc_darkmagenta, Bc_orangered4, Bc_yellowgreen, and Bc_darkorange2) were highly correlated at 0.5 hpi. Three *F. vesca* modules (Fv_yellow4, Fv_salmon4, and Fv_darkorange2) and three *B. cinerea* modules (Bc_darkslateblue, Bc_antiquewhite4, and Bc_navajowhite2) were highly correlated at 1 hpi. Two *F. vesca* modules (Fv_lightpink4 and Fv_skyblue3) and one *B. cinerea* module (Bc_mediumpurple3) were highly correlated at 3 hpi. Three *F. vesca* modules (Fv_thistle2, Fv_brown4, and Fv_honeydew1) and one *B. cinerea* module (Bc_lightcoral) were highly correlated at 9 hpi. Four *F. vesca* modules (Fv_brown, Fv_blue, Fv_plum, and Fv_palevioletred3) and four *B. cinerea* modules (Bc_darkolivegreen4, Bc_lightcyan, Bc_darkolivegreen, and Bc_darkgreen) were highly correlated at 11–12 hpi. To confirm the co-expression of individual genes, correlation coefficients were calculated between pairs of genes from highly correlated modules in *F. vesca* and *B. cinerea* (Fig. 7A). Based on this analysis, we identified 56 genes encoding secreted protein in *B. cinerea* (Fig. 7B) whose expression was highly correlated with that of 13 receptor-like genes in *F. vesca* (Fig. 7C), and we then tested the potential physical interactions of their proteins by yeast two-hybrid and BiFC assays. As shown in Fig. 8A, BcXYG1, BcPG3, and FvRLP2 do not form homodimers themselves. In the presence of the FvRLP2 protein, BcXYG1 and BcPG3 proteins interact with the FvRLP2 protein. BiFC assays further validated these interactions in tobacco leaves. Fluorescent signals confirmed that BcXYG1 and BcPG3 interacted with FvRLP2 (Fig. 8B; Fig. S7, see online supplementary material).

### Functions of *FvRLP2* in disease resistance

**Figure 9:**
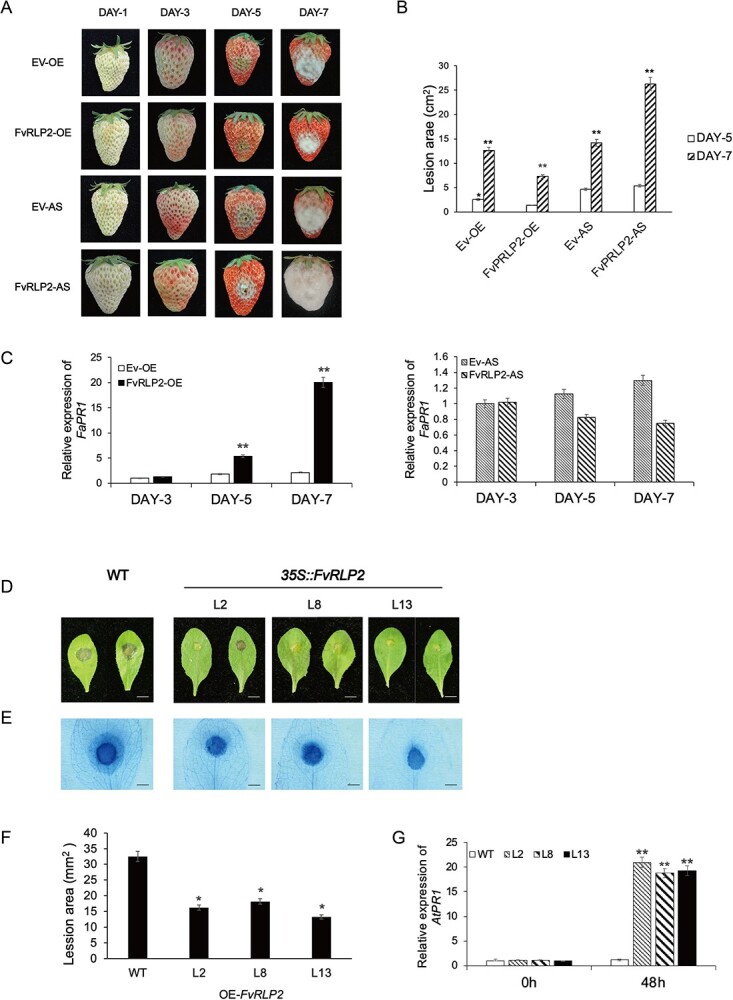
Phenotype produced by transient overexpression or silencing of *FvRLP2* in strawberry fruits and *Arabidopsis* plants. (**A**) Lesion development after inoculation of strawberry fruits with *Botryti cinerea*. DAY-1 indicated the first day after fruit were injected with *Agrobacterium* containing overexpression or silencing constructs of *FvRLP2*. DAY-3 indicated the first day after inoculation with *B. cinerea*. AS, silencing vector; EV, overexpression empty vector; OE, overexpression vector. (**B**) Measurement of lesion area using ImageJ. (*n* = 10, Student’s *t-*test, *P < 0.05 and **P < 0.01). (C) Relative expression levels of *FaPR1* in fruit from different treatments (*n* = 10, Student’s *t-*test, *P < 0.05 and **P < 0.01). (**D**) Leaf phenotypes of transgenic *Arabidopsis* overexpressing *FvRLP2* and wild-type control plants during infection by *B. cinerea*. (**E**) Trypan blue staining revealed cell death at 48 h after infection. (**F**) Measurement of lesion area using ImageJ (*n* = 10, Student’s *t-*test, *P < 0.05 and **P < 0.01). (**G**) Relative expression levels of *AtPR1* in the transgenic lines, respectively (*n* = 10, Student’s *t-*test, *P < 0.05 and **P < 0.01).

To investigate the roles of *FvRLP2* in disease resistance, we injected solutions of *Agrobacterium* containing overexpression and silencing constructs of the three genes into *F. vesca* fruit and observed lesion sizes during the resulting infection (Fig. 9A). DAY-1 was the first day after *Agrobacterium* injection, *B. cinerea* was inoculated into the fruit on DAY-3, and obvious lesions were observed on DAY-5. ImageJ measurements revealed that lesion size varied markedly among fruit from different treatments. Compared with controls, lesion areas were significantly smaller in fruit overexpressing *FvRLP2* and larger in fruit in which *FvRLP2* had been silenced (Fig. 9B). qRT-PCR analysis confirmed that the expression levels of defense-related genes *FaPR1*, *FaNPR1*, *FaWRKY29*, and *FaMAPK6* increased on the 5th and 7th days in *FvRLP2-OE* plants, especially FaPR1, which was up to 20 times higher than that of the control. (Fig. 9C; Fig. S8A, see online supplementary material).

We next performed heterologous overexpression of *FvRLP2* in *Arabidopsis* and inoculated the transgenic plants with *B. cinerea* (Fig. 9D). Trypan blue staining revealed that lesion areas were significantly smaller in plants overexpression *FvRLP2* than in control plants at the same period (Fig. 9E and F). QRT-PCR showed that the expression of *AtPR1*, *AtPR4*, *AtNPR1*, *AtWRKY29*, *AtMAPK6* genes were higher in *FvRLP2-OE* lines 3, 7, and 15 than in WT *Arabidopsis* at 48 h (Fig. 9G; Fig. S8B, see online supplementary material). These results further confirmed that *FvRLP2* played important roles in *F. vesca* resistance to *B. cinerea*.

## Discussion

Strawberry infected with gray mold lead to a decrease in photosynthesis and even plant death. Once infected by *B. cinerea*, it is difficult to eliminate, resulting in reduced fruit yield and quality [26]. After being attacked by *B. cinerea*, strawberry undergoes the stage of asymptomatic infection first, and then the disease begins to break out when the conidia of the pathogen germinate and colonize the plant surface [34]. We observed the period of lesion appearance by trypan blue staining and determined that 0–12 hpi was the early stage of strawberry infection with *B. cinerea* combined with the scanning electron microscope observation in the previous study [35].

### Defense strategies in *F. vesca*

Pathogen infection triggered a rapid plant response in plants at the initial infection stage [36, 37]. The plant cell wall and waxy cuticle constitute the first important protective barrier against pathogen attacks, which stimulates defense responses by sensing external environmental changes and transmitting signals [38]. During different types of plant–pathogen interactions, xyloglucan metabolism is regulated to defend against pathogen invasion [39, 40]. In this study, we found that there was a significant xyloglucosyl transferase (*FvH4_2g29420*, *FvH4_4g09230*, and *FvH4_3g00840*) and increased gene expression related to signal recognition and transduction (*FvH4_2g19020* and *FvH4_3g20490*) at 0.5 hpi, which then plateaued in the subsequent hours. Pattern recognition receptors for microbe-associated molecular patterns (MAMPs), such as LRR kinases and lysine motif kinase receptors, were involved in the initial perception of plant pathogen [17, 41]. For instance, LRR genes were upregulated immediately after infection. Most other genes related to pathogen recognition and signaling also responded immediately, such genes encode members of the calmodulin-binding family and NBS-LRR proteins. It has been reported that when sensing external changes, the instantaneous influx of Ca^2+^ causes calcium transients and oscillations in plants, resulting in an initial response to stimuli. Plants directly recognize effector molecules through the nucleotide-binding site NBS-LRR and TLR protein receptors or indirectly perceive the presence of effector molecules by detecting the activity of effector molecules, causing a strong immune response [42]. We speculate that this is because plants will make a rapid defense response to external stimuli and transmit the generated signals to other cells and genes after inoculation with *B. cinerea*. The number of differentially expressed genes and GO enrichment results showed that the number of differentially expressed genes increased significantly at 5 hpi, and GO entries related to ‘defense response’ and ‘response to abiotic stress’ were enriched, indicating that strawberry leaf epidermis began to perceive the presence of pathogen and began to resist pathogen invasion. TFs act as transcriptional activators or repressors, regulating immune-related genes related to hormone signaling pathways and pathogen-associated molecular patterns to enhance plant defenses [43]. Perception of PAMPs or pathogen effectors was transduced by plant MAPK signaling cascades in which WRKY TFs were involved [44]. NB-LRR gene *OsRLR1* has been reported to interact with OsWRKY19 to mediate plant defense responses in rice [45]. We focused on 22 TF families that were highly expressed at the early stage of infection. For example, the bHLH family genes were mainly expressed at 0.5 hpi, the C2H2 and C3H family genes were mainly expressed at 7 hpi, and the WRKY and ERF gene families were mainly expressed at 10–11 hpi. Hormonal signaling pathways in plants form a complex regulatory network that could attenuate pathogen virulence. Plant hormones such as jasmonic acid, salicylic acid, ethylene, auxin, cytokinin, and gibberellin have been found to play an important role in coordinating intercellular communication during pathogen attack perception [46, 47]. We observed that the genes involved in the hormone signal transduction pathways were highly expressed at different treatment points. In the process of defending against infection by *B. cinerea*, ET has been reported to act as a modulator to positively regulate the SA response gene PR1, thereby interfering with the antagonistic interaction between SA and JA [48]. The genes related to SA, JA, and ET pathways were upregulated after 7 hpi in our study (Fig. 4). Identification of these genes and gene families that respond quickly to *B. cinerea* infection lay a foundation for continued study of early defense responses in *F. vesca*.

### Pathogenicity of *B. cinerea*

To infect plant host tissues, *B. cinerea* has evolved a range of abilities that enable it to overcome plant resistance [26]. *B. cinerea* may have initiated growth, development, and pathogenic attack when it sensed the presence of the host, especially at 0–3 hpi, when expression data suggested that metabolic activities and biosynthetic processes were strongly induced (Fig. 2B). CWDEs were important for the pathogen to destroy plant cell walls without penetrating structures or in the late stages of necrotic pathogen invasion. When *B. cinerea* senses the presence of hosts, it secretes a large number of cell wall degrading enzymes to destroy plant cell walls and use them as a source of nutrition [49]. Previous reports have shown that massive upregulation of carbohydrate-active enzyme (CAZyme)-encoding genes were involved in the infiltration, colonization, and spread of pathogenic during infection [50, 51]. We found that *B. cinerea* began to initiate pathogenic gene functions to attack the host by increased expression of CAZymes, SOD, and GST-related genes (Fig. 5). Notably, two endoglucanase genes (*BCIN_02g01960* and *BCIN_03g01610*) were upregulated at 0.5 hpi and 1 hpi, respectively. Autophagy plays a wide range of biological roles in eukaryotes by maintaining cell homeostasis. In the early stages of infection, autophagy enables *B. cinerea* to respond to nutrient-limiting states [52]. Additionally, *BcAtg2*-mediated autophagy has been reported to be necessary for the pathogenicity of *B. cinerea* [53]. In this study, two genes encoding autophagic proteins (*BCIN_09g05260* and *BCIN_08g04190*) were also highly expressed at 0.5 hpi. In addition, fungal effectors promote pathogen virulence by inhibiting pattern-triggered immunity (PTI) and effector-triggered immunity (ETI) signaling [54]. Several studies have demonstrated that *B. cinerea* secreted proteins could induce strong necrosis and host resistance [15, 16, 55]. In the present study, 251 secrete-related genes from *B. cinerea* were upregulated during the process of infection, and 15.93% (40/251) of them were differentially expressed in at least 10 infection stages (Fig. 5). This evidence suggested that *B. cinerea* attacks the immune system of strawberry through multiple pathways, ultimately resulting in strawberry necrosis.

### Interactions between *F. vesca* and *B. cinerea*

To further characterize the mechanisms of interaction between *F. vesca* and *B. cinerea* during the early infection, we constructed a co-expression network using whole-genome expression data by WGCNA. This approach analyzes the expression patterns of genes in multiple samples and has been widely used in the study of plant-pathogen interactions in horticultural plants [25, 56, 57]. In this study, there were corresponding positive correlation expression modules in each treatment stage of woodland strawberry and *B. cinerea* (P < 0.01), indicating that there was specific gene expression in each treatment stage. The KEGG enrichment of the co-expression module showed that the pathways related to ‘cutin, cork, and wax biosynthesis’ and ‘plant hormone signal transduction’ were enriched at 0.5 hpi in woodland strawberry, indicating that the wax layer of the plant epidermis perceives the initial changes of external stimuli and responds quickly. At 6 hpi, the pathways related to starch and sucrose metabolism were enriched in both *B. cinerea* and strawberry, indicating that sugar was needed as an energy source during pathogen colonization and host defense. At 8 hpi, the glycolysis pathway was enriched in strawberry, which also proved that the molecular dialogue between host and *B. cinerea* caused significant changes in the host metabolism [58]. In addition, autophagy pathways were enriched in strawberries at 11 hpi, and reverse genetics studies have shown that autophagy plays a positive role in plant defense against *B. cinerea*. This result indicated that plants also resist the attack of *B. cinerea* in a variety of ways [59]. Seventeen gene modules in *F. vesca* and 15 gene modules in *B. cinerea* were identified as highly correlated at different treatment periods. A large number of interplay genes were identified in highly correlated modules in *F. vesca* and *B. cinerea*. This result suggested that when preparing for their arms race, pathogen and plants not only rely on their own systems of attack and defense but also may target the main forces of the other side for disruption. Several previous studies have reported that *B. cinerea* secreted proteins impair plant defense systems by interacting with key plant resistance factors [55, 60]. On the basis of WGCNA results and Y2H and BiFC assays, we found that the *B. cinerea* proteins BcPG3 and BcXYG1 interacted with FvRLP2 protein in strawberry. BcPG3 and BcXYG1 were cell death inducing proteins that have previously been shown to trigger cell death in *N. benthamiana* [15, 16]. Our results also suggested that these *B. cinerea* secreted proteins interact with strawberry proteins and may ultimately lead to necrosis; the mechanisms that link these phenomena warrant further study.

### *FvRLP2* enhances *F. vesca* resistance to *B. cinerea*

To investigate the strategy of *B. cinerea* secreted proteins, we examined the roles of *FvRLP2* in plant disease resistance. Transcriptome data showed that receptor-like proteins responded to *B. cinerea* infection in strawberry leaves (Fig. 4). *FvRLP2* (*FvH4_3g39490*), *FvRLP6* (*FvH4_2g05950*), *FvRLP7* (*FvH4_6g11660* and *FvH4_6g21370*) were highly expressed between 10–11 hpi. Previous studies have shown that receptor proteins play a crucial role in plant immunity. As pattern recognition receptors, receptor proteins can specifically recognize the presence of pathogen to induce plant defense [61]. Here, transient expression assays in strawberry fruits and transgenic analysis in *Arabidopsis* confirmed that *FvRLP2* had a positive effect on the defense response to *B. cinerea*. Functional study of these genes thus revealed that disease resistance genes in strawberry target key pathogenic factors in *B. cinerea* in order to defend against the pathogen, providing a new direction for defense against *B. cinerea*.

## Conclusion

Collectively, high-resolution time series dual RNA-seq data provided strong evidence for a dynamic arms race in strawberry resistance to *B. cinerea* infection within 0–12 hpi. The stress response occurred when strawberry leaves were stressed by *B. cinerea* at 0.5 hpi. Pathways related to keratin, cork, and wax biosynthesis and plant hormone signal transduction were enriched at 0.5 hpi in woodland strawberry, indicating that the wax layer of the plant epidermis senses the initial changes of external stimuli and responds quickly. With the extension of treatment time, it has been alleviated. At this time, the germinated hyphae of *B. cinerea* conidia continued to grow, accompanied by high expression of pathogenic genes. For example, the related pathways such as cellular protein metabolism, amide metabolism, amide biosynthesis, and organic nitrogen biosynthesis were enriched at 0.5–6 hpi in *B. cinerea*. The GO terms related to ‘defense response’ and ‘biological stimulation response’ in woodland strawberry were enriched at 5–7 hpi, indicating that it was too late for strawberry to initiate defense response after realizing the pathogenicity and hyphal formation of *B. cinerea*. WGCNA results suggested an interaction between the genes of strawberry and *B. cinerea* in the early infection process. Y2H and BiFC assays demonstrated that the strawberry disease-resistance proteins FvRLP2 interacted with the pathogenic factors BcXYG1 and BcPG3 of *B. cinerea* to defend against each other’s attack. These results provided new insights into the early stages of plant defense against a fungal pathogen and could provide a basis for the development and cultivation of new disease-resistant strawberry varieties.

## Materials and methods

### Preparation of plant material and *B. cinerea* treatment

The vegetatively-propagated *F. vesca* (‘Hawaii4’) was grown in a greenhouse at Nanjing Agricultural University (Nanjing, Jiangsu Province, China). The temperature and photoperiod were controlled at 25°C/16 h light and 22°C/8 h dark. Mycelia of *B. cinerea* (Bc05.10) were grown on CM agar plates as previously described [62]. After 3 weeks of culture in the dark, spores were collected and resuspended to 5 × 10^6^ spores/ml in SMB buffer (10 g/L mycological peptone, 40 g/L maltose). A total of 140 strawberry plants with robust growth were selected and sprayed with the conidial suspension of *B. cinerea*. SMB suspension includes conidia as the control for *B. cinerea* (Mock). The strawberry was treated with 0 hpi as the control for *F. vesca* (Mock). All plants were placed in a light incubator at 22°C and >90% humidity to provide suitable living conditions for *B. cinerea*.

### Collection of plant and pathogen samples for RNA extraction

After spraying, the 5th and 6th leaves of 10 treated strawberry plants were collected at 0, 0.5, 1, 2, 3, 4, 5, 6, 7, 8, 9, 10, 11, and 12 h. Three leaves from different plants were pooled as one replicate, and three biological replicates were collected for each treatment and time point. The spores were collected as the control sample for *B. cinerea*. All collected samples were stored at −80°C for RNA extraction.

A Plant Total RNA Isolation Kit Plus (Foregene, Chengdu, China) was used to extract total RNA (plant and fungal) from the leaf samples. Total RNA was extracted from the *B. cinerea* control samples using the TRIzol reagent (Invitrogen, Carlsbad, California USA). The OD260/280 value was confirmed to be between 1.8 and 2.2 using a NanoDrop spectrophotometer (Thermo Scientific), the RNA purity was determined by 1% agarose gel electrophoresis, and the RNA integrity was assessed with an Agilent 2100 Bioanalyzer.

### RNA-seq library construction and data analysis

Transcriptome sequencing was performed on the Illumina Novaseq 6000 platform to generate 150 bp/150 bp paired-end reads. After adaptor removal and quality control, clean reads were mapped to the reference genomes of *F. vesca* (ftp.bioinfo.wsu.edu/species/Fragaria_vesca/Fvesca-genome.v4.0.a1) [27] and *B. cinerea* (Bc05.10) (http://fungi.ensembl.org/Botrytis_cinerea) [28] using HISAT2 v2.0.4. Gene expression levels were calculated as TPM (transcripts per million reads). Differentially expressed genes (DEGs) between treated and control samples at each time point were identified using the DESeq R package (1.18.0) based on a false discovery rate ≤0.05 and a |log_2_(fold change)| ≥1. The clusterProfiler R package was used to identify Gene Ontology (GO) and Kyoto Encyclopedia of Genes and Genomes (KEGG) terms enriched in the differentially expressed genes (Padj <0.05).

### Co-expression network analysis and expression profiling of DEGs

The WGCNA R package (v1.70) in R was used to perform weighted gene co-expression network analysis (WGCNA) with default parameters [63, 64]. All genes from *F. vesca* and *B. cinerea* were used for network construction. For the standard WGCNA network of *F. vesca*, the soft threshold was set to 9, and the modulesize was 20. For the standard WGCNA network of *B. cinerea*, the soft threshold was set to 7, and the modulesize was 5. Correlations between modules from *F. vesca* and *B. cinerea* were calculated using the ‘cor’ function in R (r ≥ 0.8, *P*-value <0.05). The network of highly correlated genes in *F. vesca* and *B. cinerea* was visualized using Cytoscape [65] (v3.9.1, USA) (r ≥ 0.8, *P*-value <0.05). A trend analysis of DEGs expression profiles was performed using Short Time-series Expression Miner (STEM) software [66], and genes that showed similar patterns of expression through time were grouped into STEM profiles (*P* ≤ 0.01).

### Quantitative RT-PCR

RNA from all samples was reverse transcribed to obtain cDNA using the Prime Script RT reagent kit with gDNA eraser (Takara, Dalian, China), and qRT-PCR was performed using SYBR Green II (Takara) according to the instructions of the LightCycler 480 system (Roche, Switzerland). All primers used were listed in Table S8 (see online supplementary material). Relative gene expression was calculated by the 2^−ΔΔCt^ method using the *FvGAPDH2* gene as the reference gene in strawberry and the *Bcactin* gene as the reference gene in *B. cinerea*.

### Physiological analysis

The leaf tissue treated at each time period was selected and frozen at −80°C for use. Contents of H_2_O_2_ and malondialdehyde (MDA) and activities of peroxidase (POD) and catalase (CAT) were measured according to the protocols of the relevant assay kits (Solarbio, China) using a Cytation3 plate reader (BioTek).

### Yeast two-hybrid assays

Y2H Gold cells were used for yeast two-hybrid screens. The full-length coding sequences of *BcPG3* (*BCIN_04g04930*) and *BcXYG1* (*BCIN_03g03630*) were cloned into the pGBKT_7_ vector, the CDS of *FvRLP2* (*FvH4_3g39490*) was amplified and inserted into the pGADT_7_ vector. The vector of pGAD53m and pHIS2-P53 were co-transformed as positive controls, while the vector of pGAD53m and pHIS2 were co-transformed as negative controls. The LiCl-PEG method was used for yeast transformation as described previously [67]. Inoculate yeast strains with different combinations of plasmids on synthetic dropout media lacking Trp and Leu (SD/−Trp/−Leu) solid medium and incubate at 30°C for 3 days. Extract and transfer the single colonies onto synthetic dropout (SD) media lacking Ade, His, Trp, and Leu (SD/−Ade-His-Trp-Leu) and SD/−Ade-His-Trp-Leu supplemented with 20 mg/ml X-⍺-Gal was used to detect the possible interactions. All transformations and screenings were performed three times. The primers used for vector construction are listed in Table S8 (see online supplementary material).

### Bimolecular fluorescent complementation (BiFC) assay

The CDS sequences of *BcXYG1*, *BcPG3*, and *FvRLP2* were amplified and inserted into the pSPYCE (M) and pSPYNE 173, respectively. The recombinant plasmids and controls were transferred into *Agrobacterium* GV3101 and cultured in yeast extract peptone (YEP) medium at 28°C. The bacterial solutions were mixed in proportion and injected into two-month-old *Nicotiana benthamiana* leaves, the resulting fluorescence was observed by confocal laser scanning microscopy (LSM 800, Zeiss, Germany).

### Expression by the transient transformation in strawberry fruit

Construct the CDS sequence of the *FvRLP2* gene onto the PJ5s vector or the pFGC5941 vector, respectively. The PJ35s vector containing the 35S promoter and the RNAi vector pFGC5941 were used as control groups for overexpression and RNAi treatment. Agrobacterium-mediated transient transformation of strawberry was performed as described previously [68]. In brief, *Agrobacteria* containing *FvRLP2-OE*, and *FvRLP2-RNAi* plasmids were cultured at 28°C for 16 hours. When the OD_600_ values were 0.6–0.8, they were centrifuged and resuspended in buffer (10 mM MgCl_2_, 10 mM MES, and 40 μM Acetosyringone). The fruit during the large green fruit stage (about 30 days after flowering) was selected for injection. The bacterial solutions were injected from the bottom into individual strawberry fruit. Three days after infection, 5 × 10^6^ spores/ml conidia of *B. cinerea* were inoculated onto the fruit, 30 strawberry fruits were injected in each treatment, and the occurrence of lesions was observed over the following days.

### Phenotype analysis of transgenic Arabidopsis

We carried out transgenic experiments to verify the functions of selected disease-resistance genes in *Arabidopsis*. The CDS sequence of FvRLP2 was constructed into the PJ5s vector to form the *FvRLP2-OE* plasmid. *Agrobacterium* carrying *FvRLP2-OE* were transferred into *Arabidopsis* by the floral dip method [69]. The plants were grown to maturity, and their seeds were harvested. After seed harvest, 50 mg/ml hygromycin was used for selection, and the T3 generation of transgenic *Arabidopsis* was used for phenotypic analysis. A total of 14 *FvRLP2* overexpression lines were obtained, respectively. The rosette leaves of *Arabidopsis* were inoculated with *B. cinerea* conidia. Wild-type *Arabidopsis* was used as a control, and the changes in the resulting lesions were observed by trypan blue staining [70].

## Supplementary Material

Web_Material_uhad225
