# Supplementary figures and images for: The dynamic arms race during the early invasion of woodland strawberry by Botrytis cinerea revealed by dual dense high-resolution RNA-seq analyses
Source: Hortic Res. 2023 Nov 9;10(12):uhad225. doi: 10.1093/hr/uhad225 (PMC10745266; doi:10.1093/hr/uhad225)

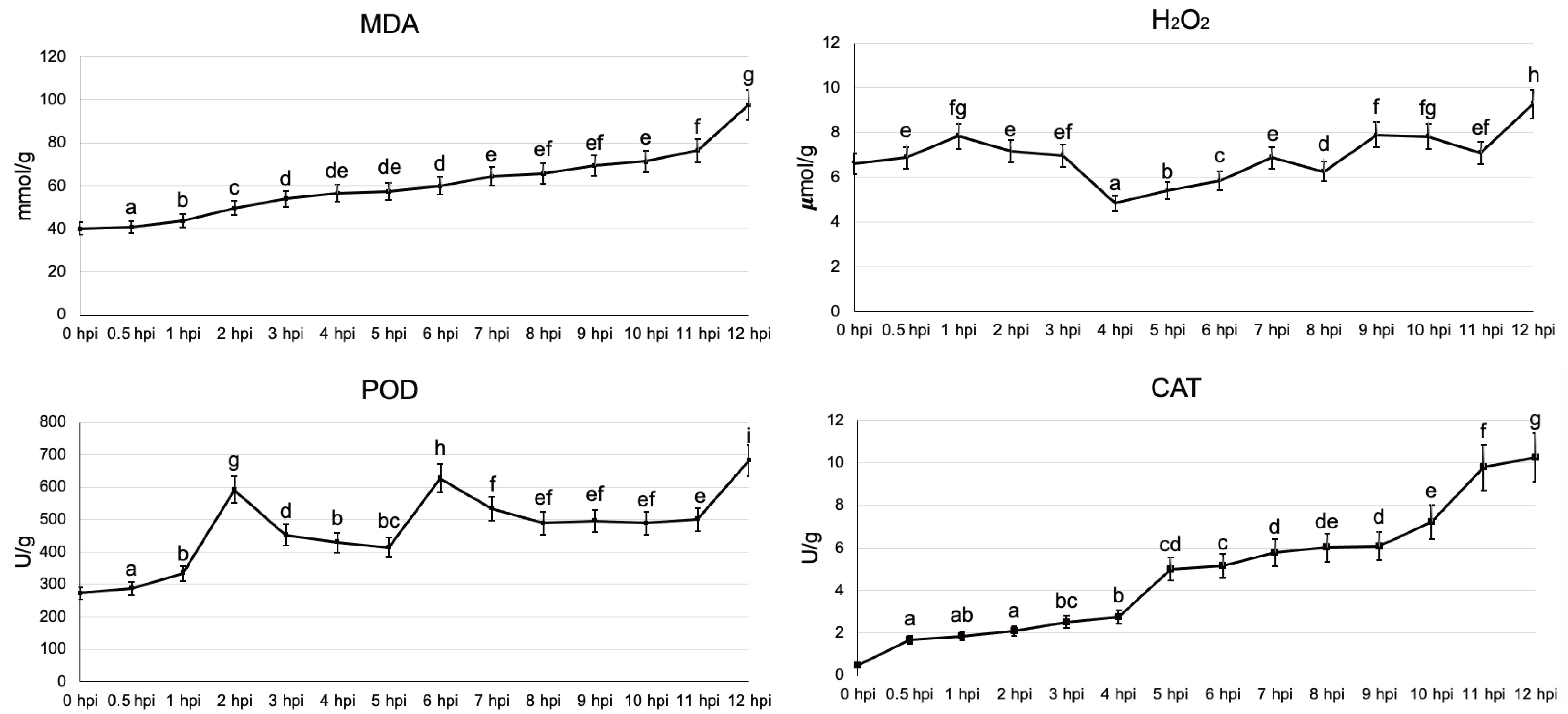

Supplement: Web_Material_uhad225 [file web_material_uhad225.zip › Fig.S1.jpg]

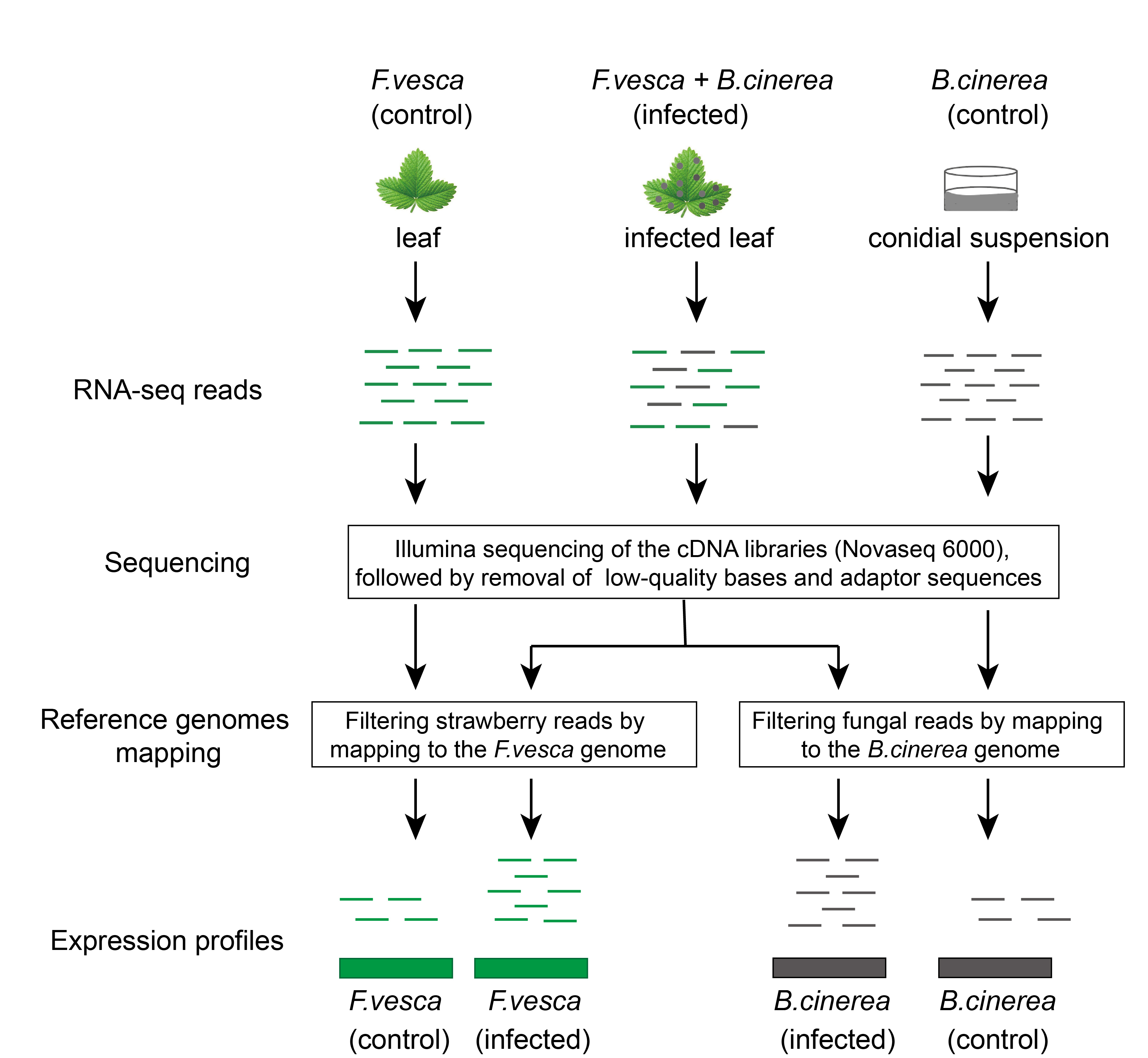

Supplement: Web_Material_uhad225 [file web_material_uhad225.zip › Fig.S2.jpg]

A

**FvPR2**  
(FvH4\_2g18200)

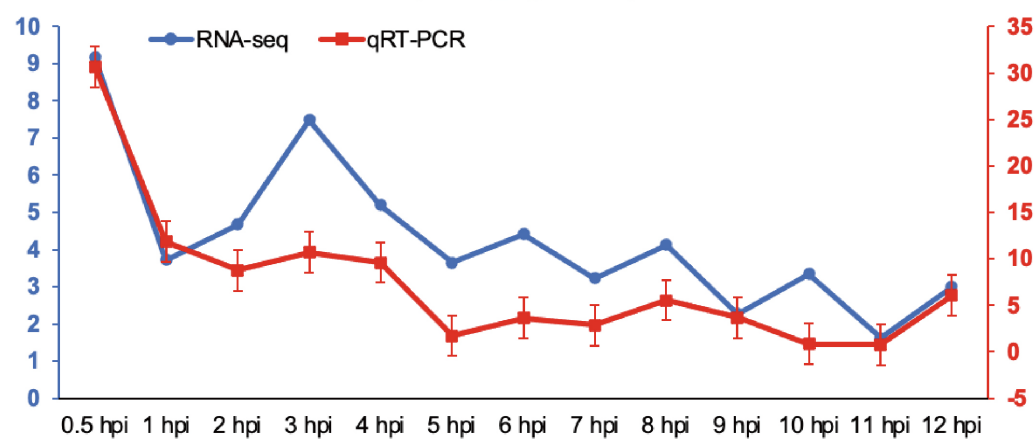

**FvRLP2**  
(FvH4\_3g39490)

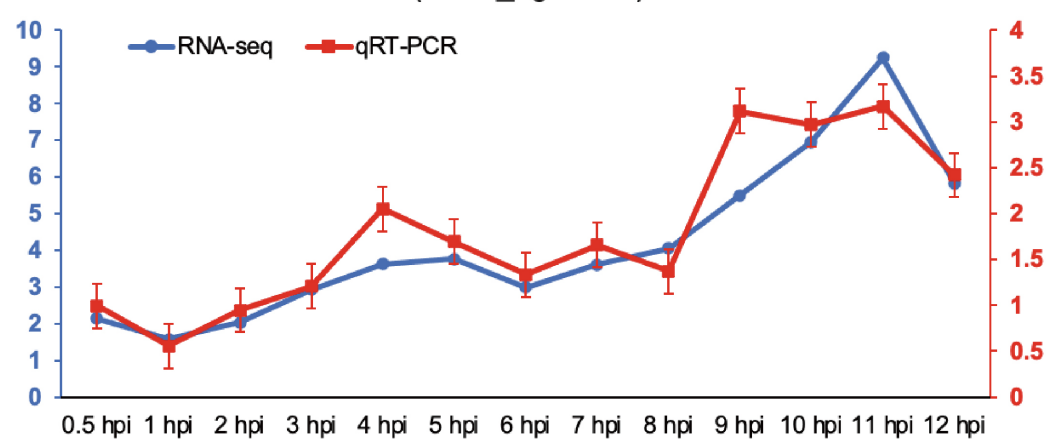

**FvRLP7**  
(FvH4\_6g11660)

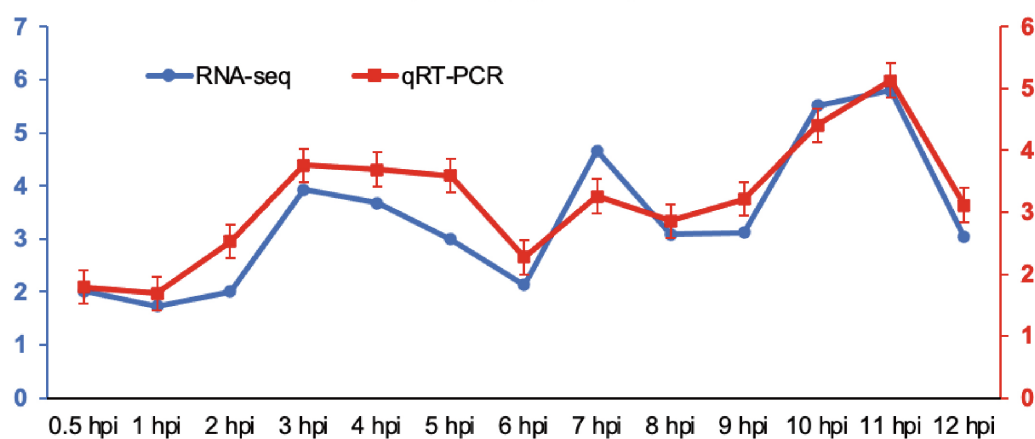

**FvWRKY**  
(FvH4\_6g10510)

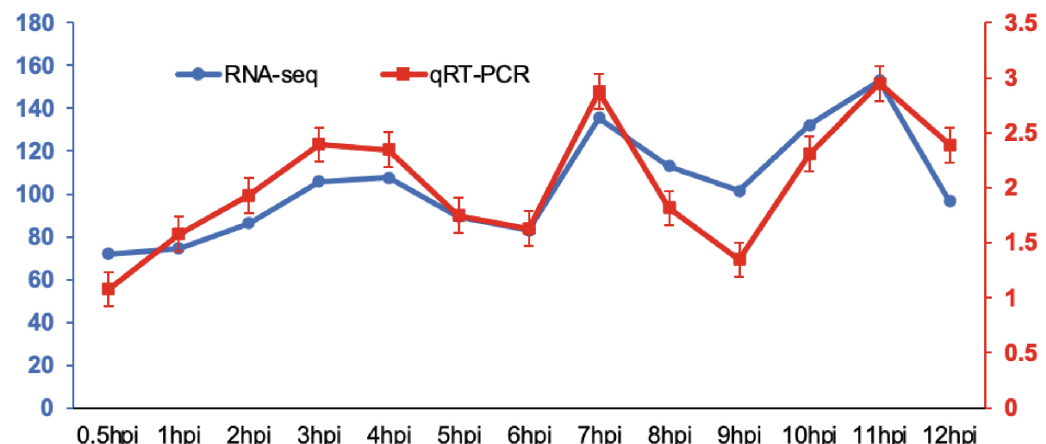

B

**BcXYG1**  
(BCIN\_03g03630)

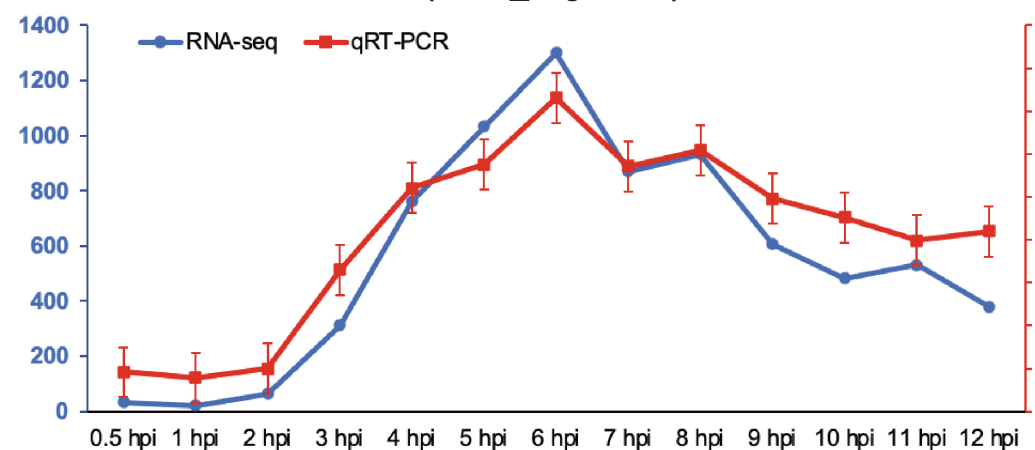

**BcNEP1**  
(BCIN\_06g06720)

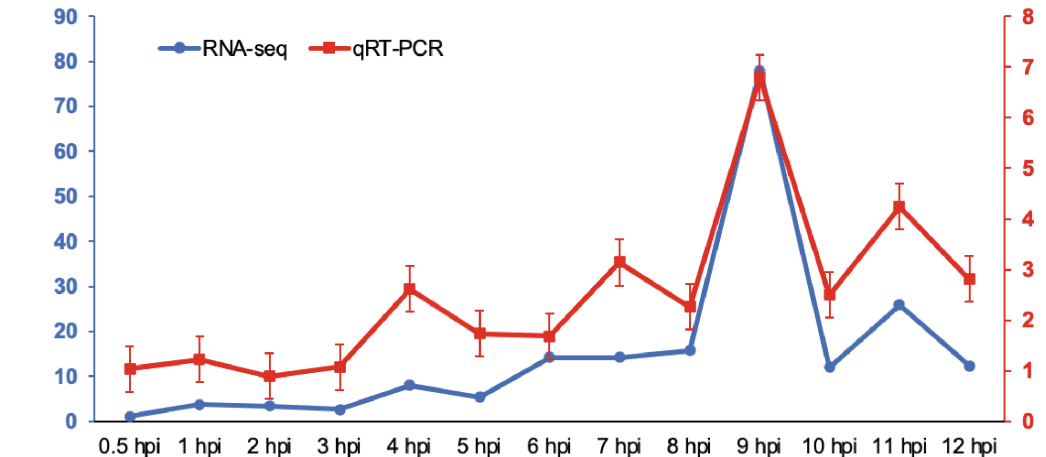

**BcXyn11A**  
(BCIN\_03g00480)

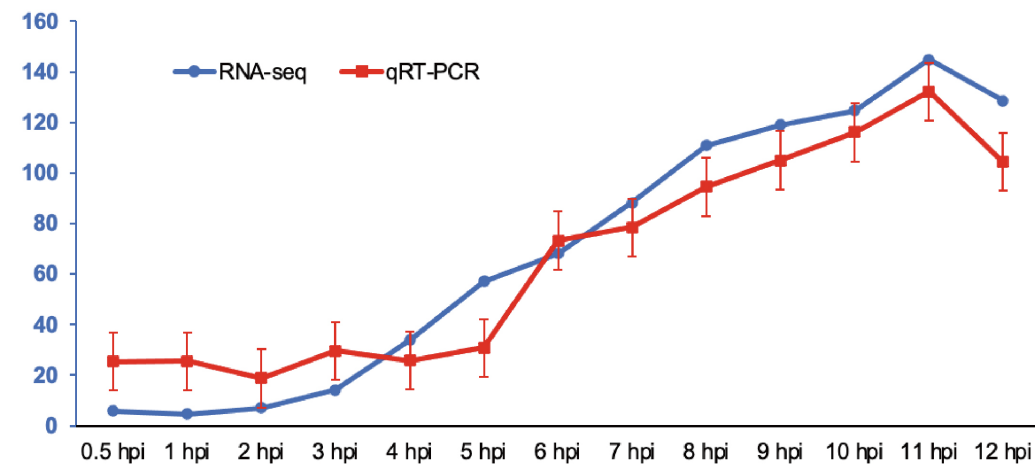

**BcIEB1**  
(BCIN\_15g00100)

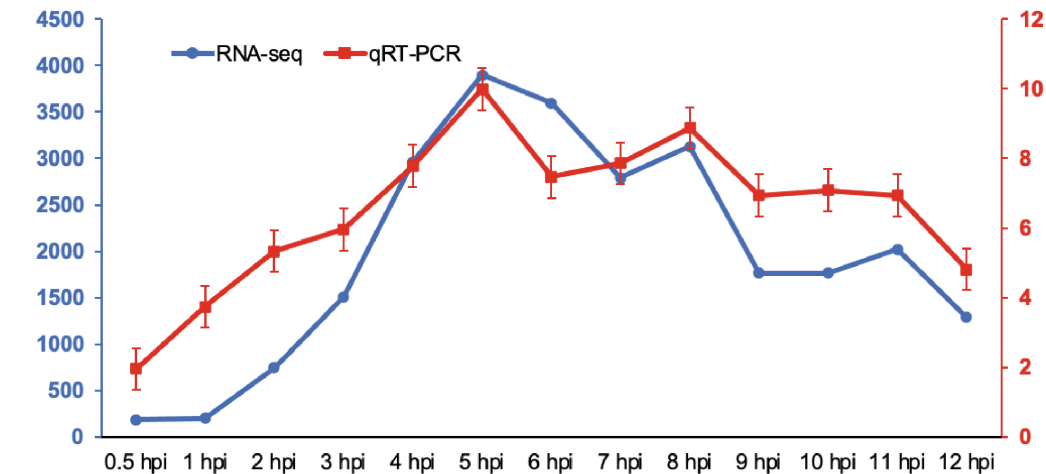

Supplement: Web_Material_uhad225 [file web_material_uhad225.zip › Fig.S3.pdf]

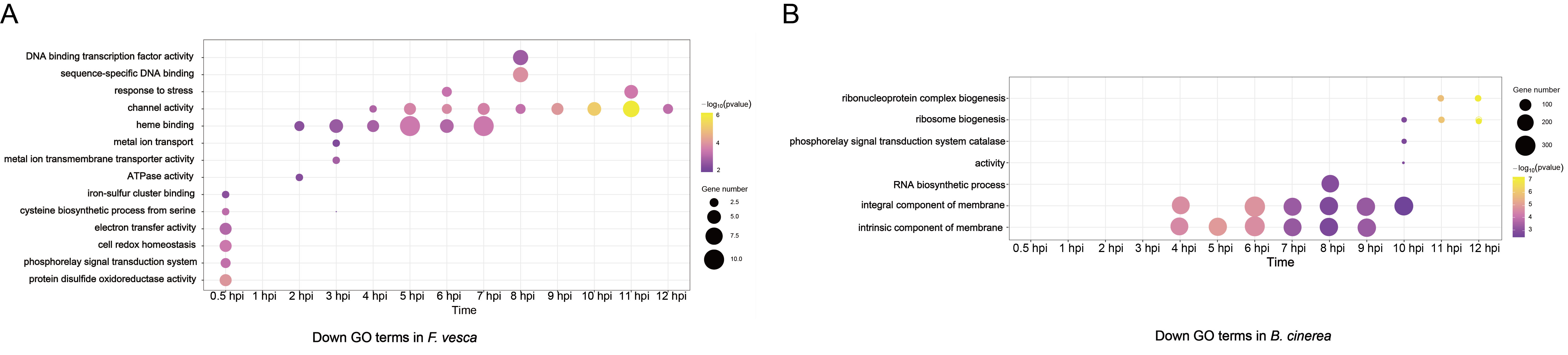

Supplement: Web_Material_uhad225 [file web_material_uhad225.zip › Fig.S4.jpg]

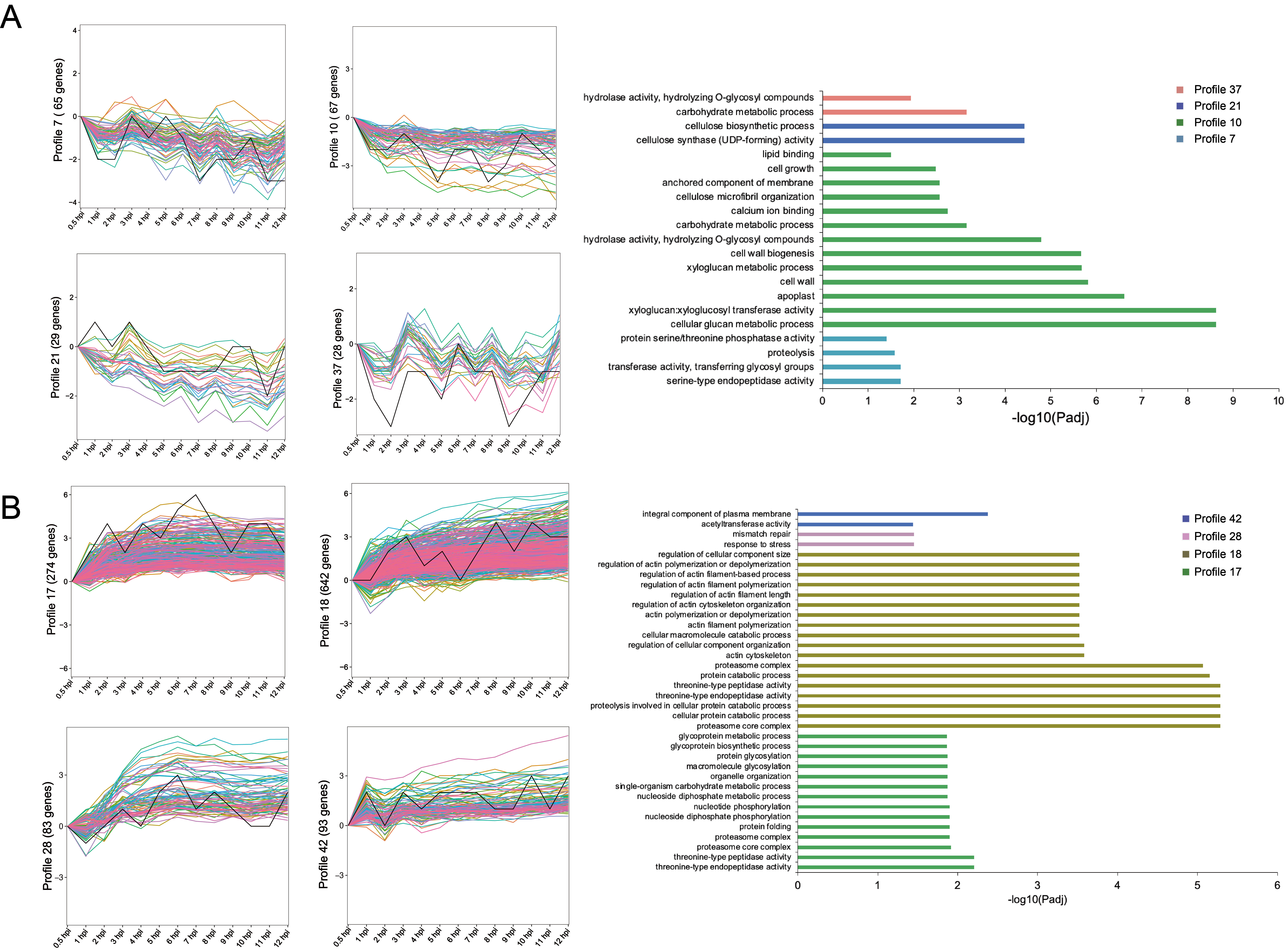

Supplement: Web_Material_uhad225 [file web_material_uhad225.zip › Fig.S5.jpg]

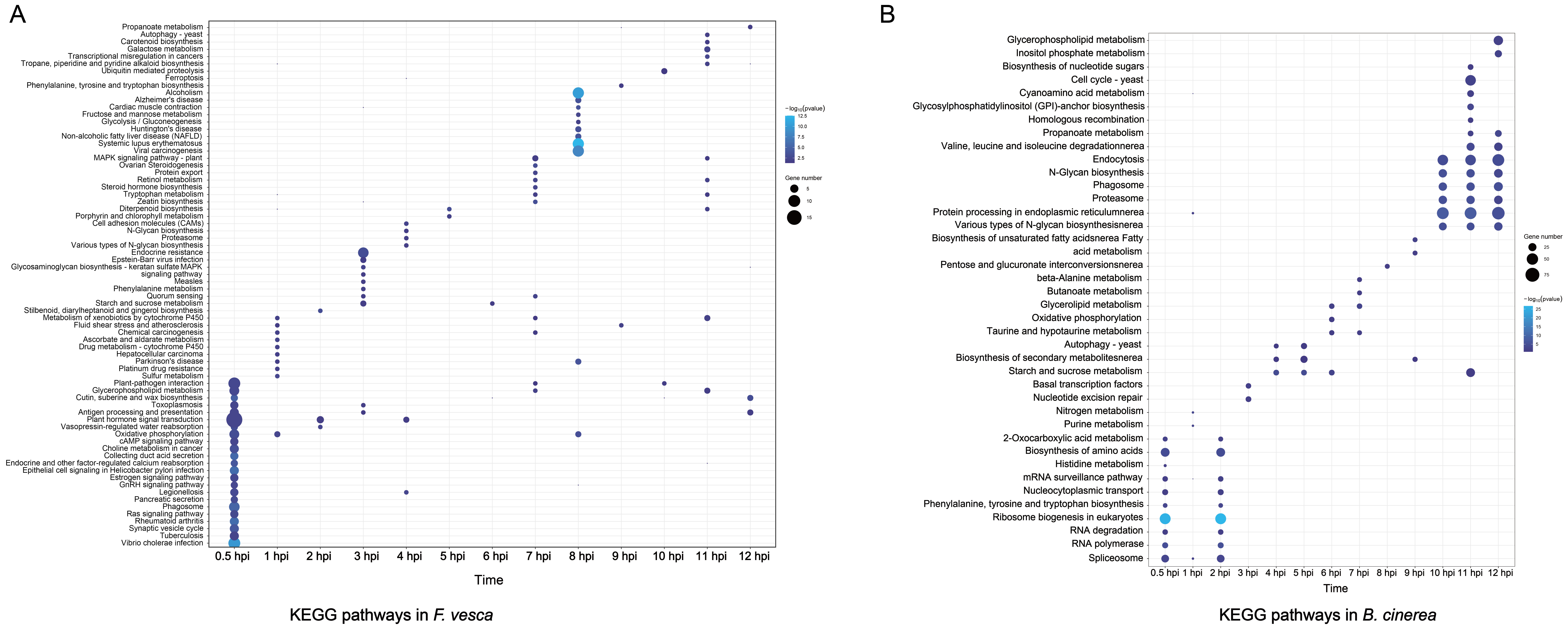

Supplement: Web_Material_uhad225 [file web_material_uhad225.zip › Fig.S6.jpg]

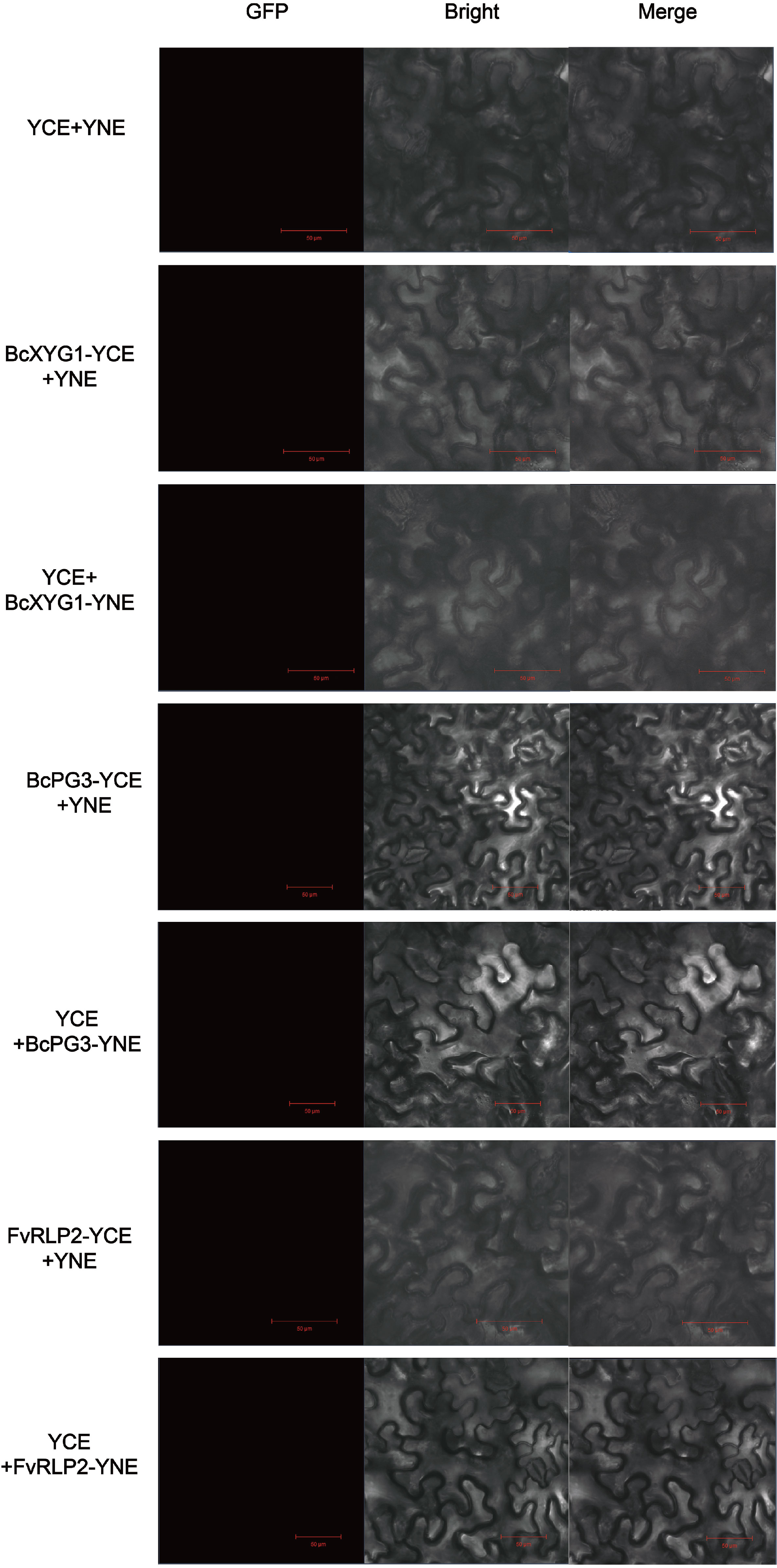

Supplement: Web_Material_uhad225 [file web_material_uhad225.zip › Fig.S7.jpg]

A

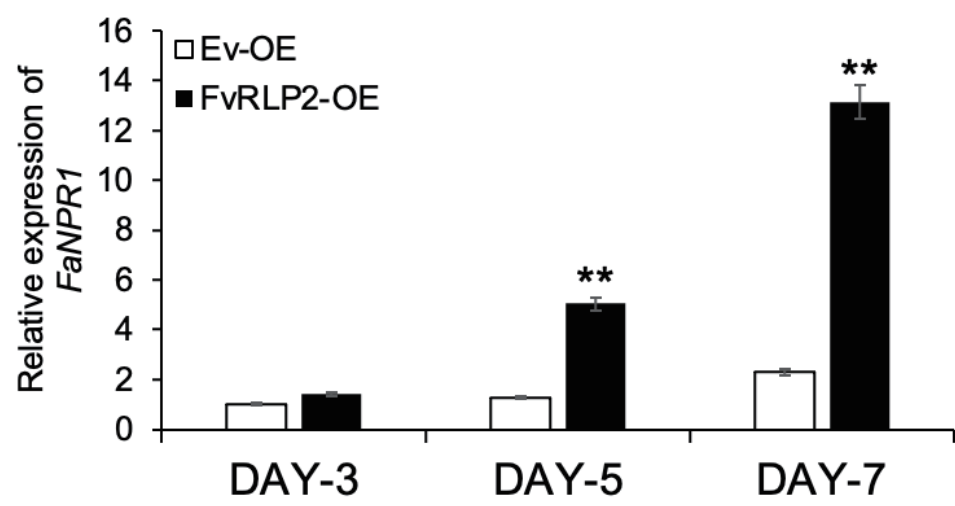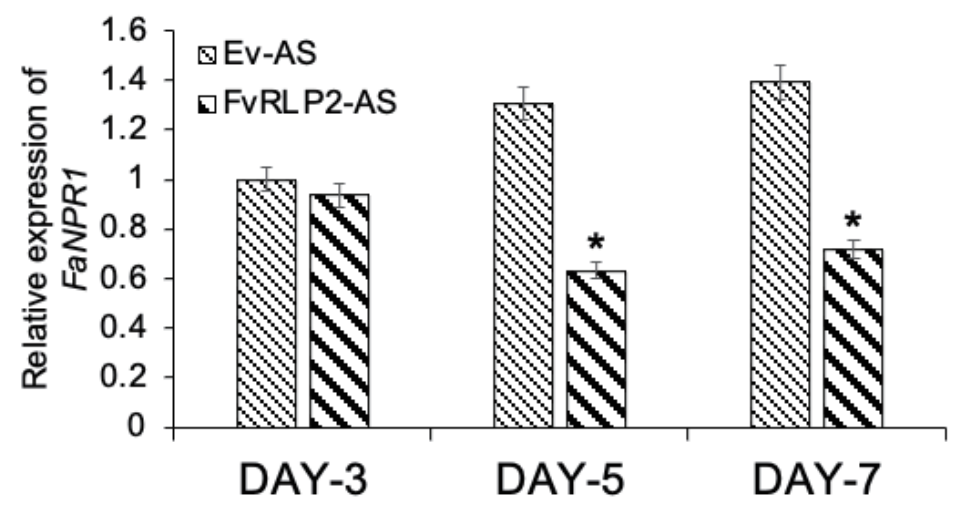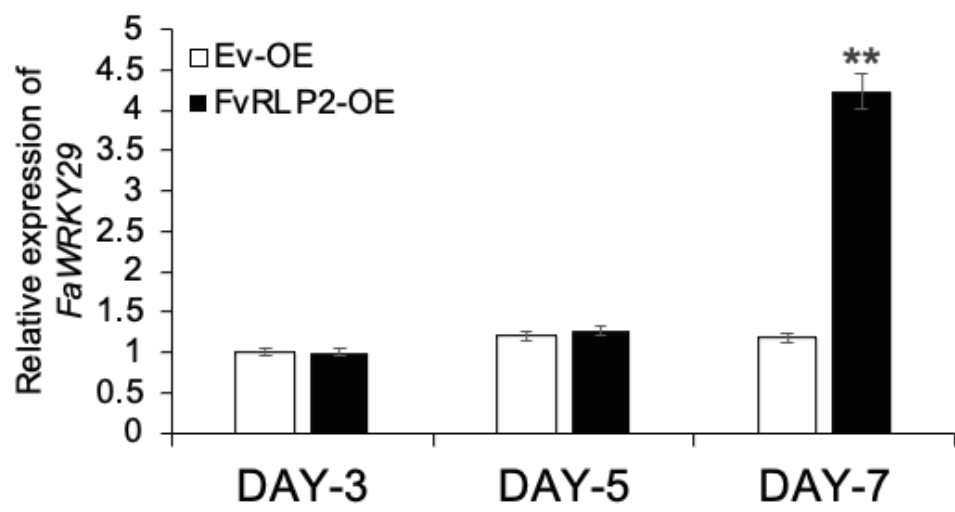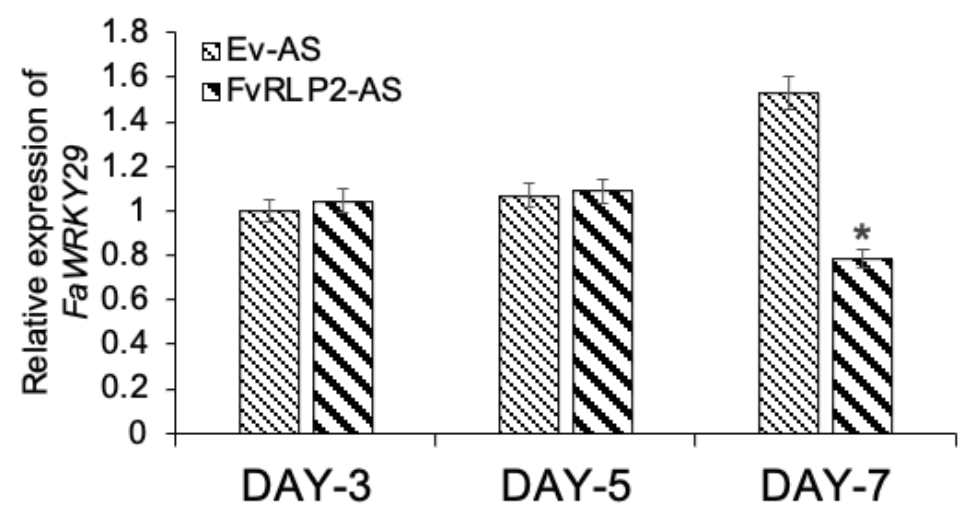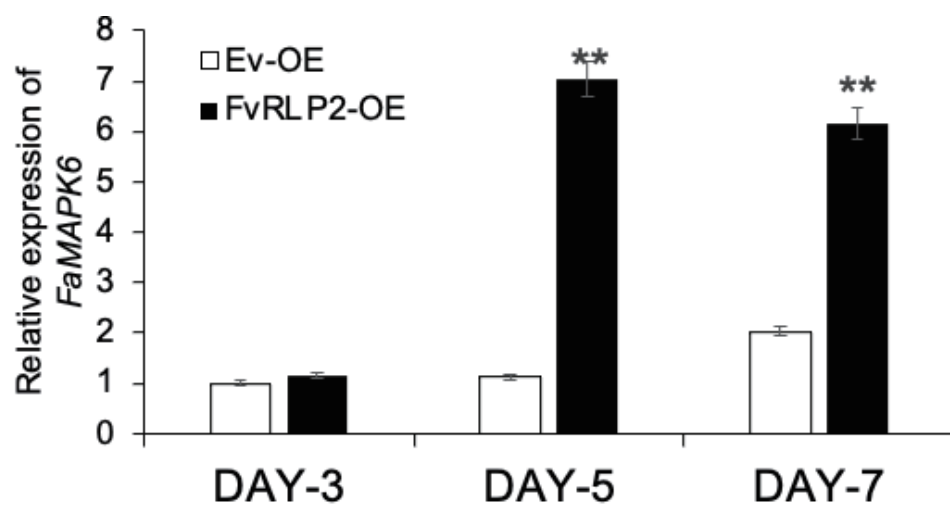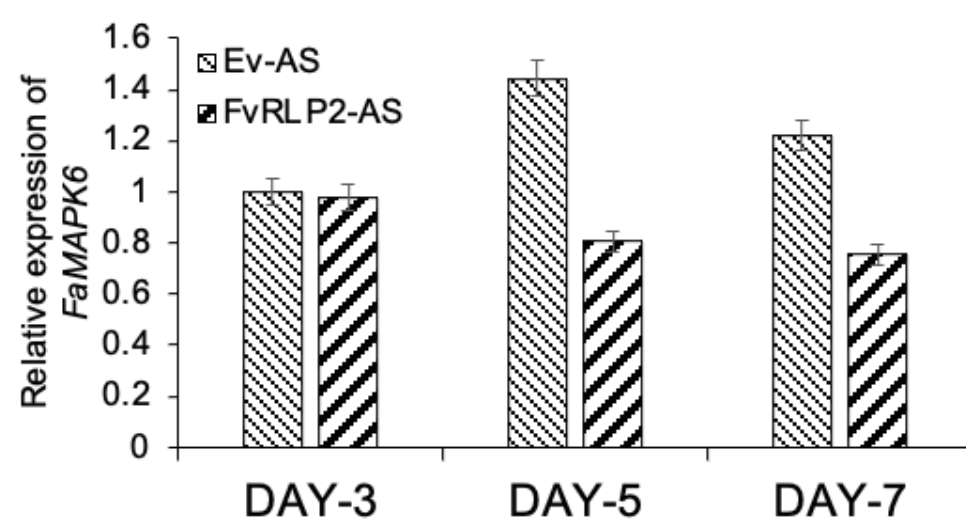

B

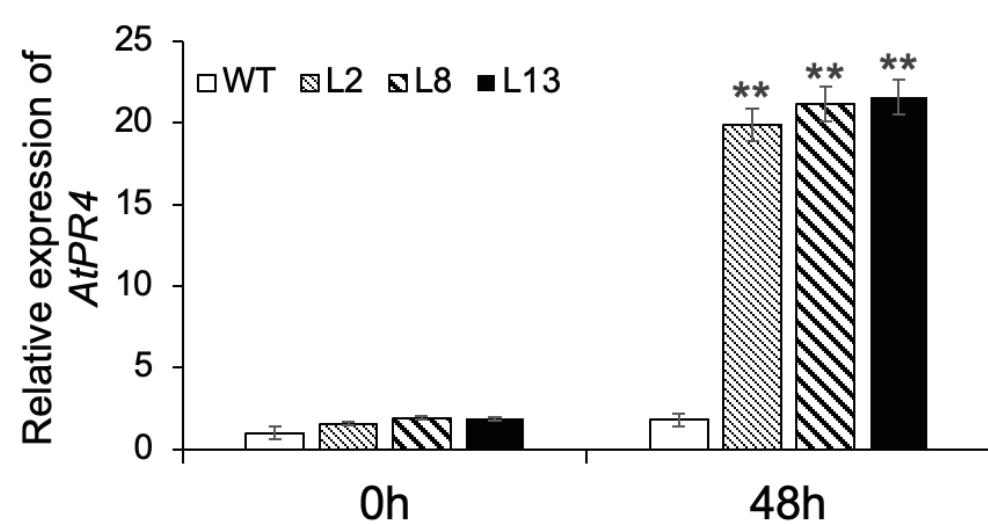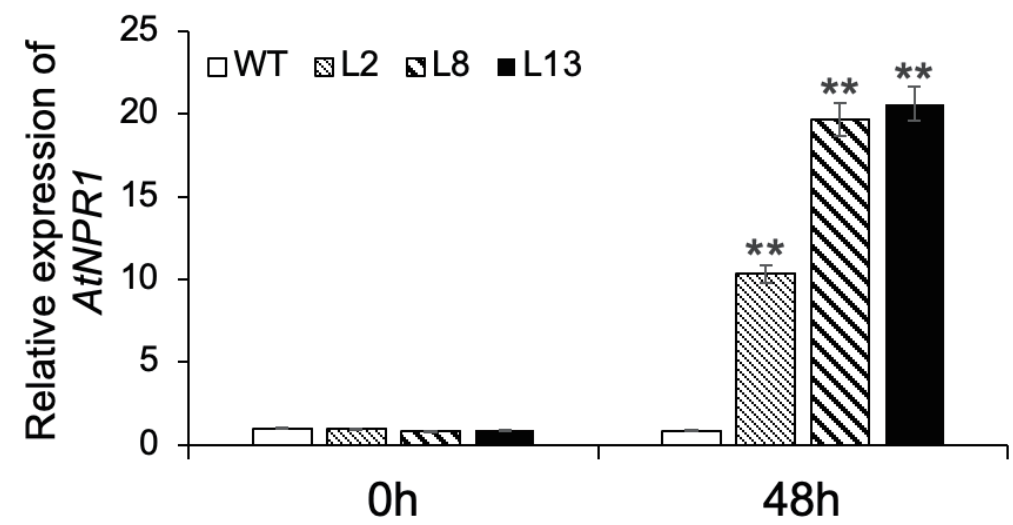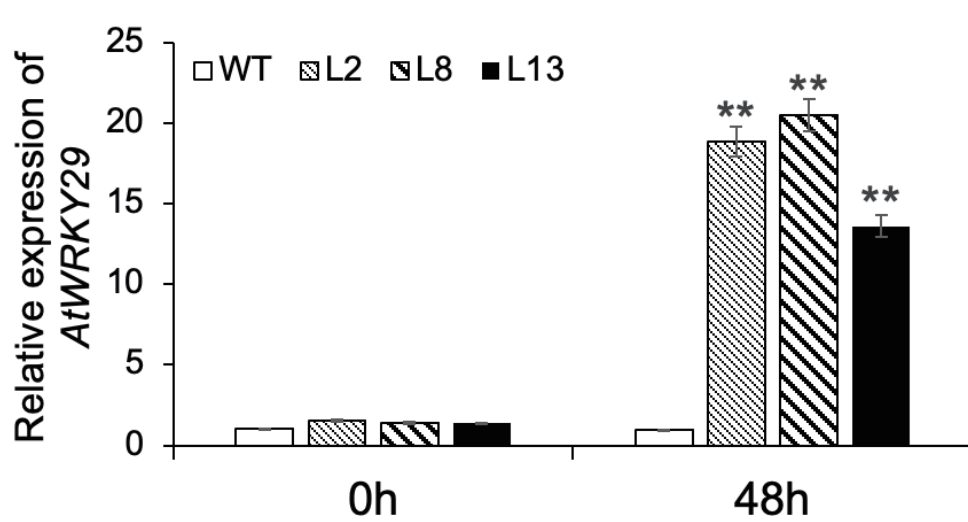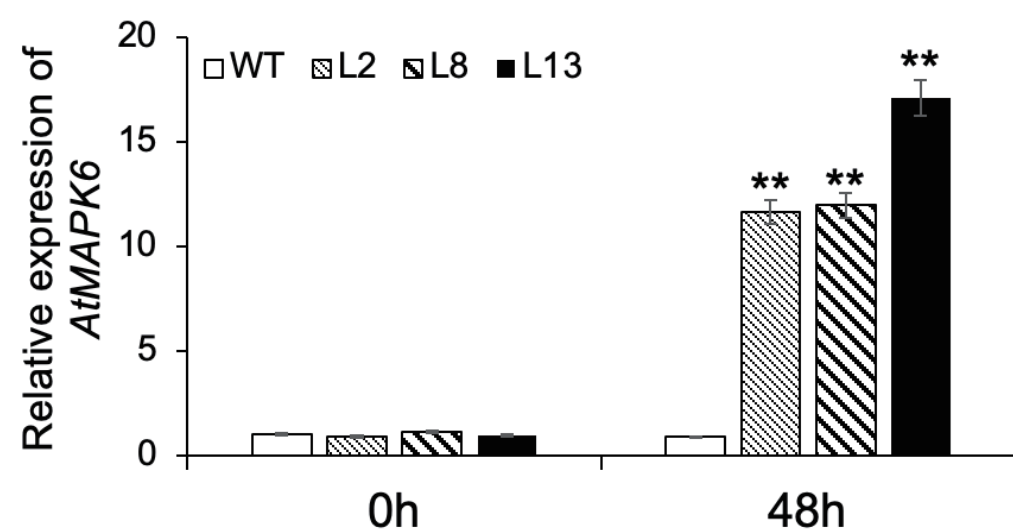

Supplement: Web_Material_uhad225 [file web_material_uhad225.zip › Fig.S8.pdf]
